# Supplementary material for: Endoplasmic reticulum stress actively suppresses hepatic molecular identity in damaged liver
Source: Mol Syst Biol. 2020 May 14;16(5):e9156. doi: 10.15252/msb.20199156 (PMC7224309; doi:10.15252/msb.20199156)
Supplement: Supplementary file 1 — Appendix [file MSB-16-e9156-s001.pdf]

APPENDIX Table of Contents

Appendix Figure S1. Additional characterization of transcriptomic changes induced by acute ERS - related to Fig.1.

Appendix Figure S2. All model profiles with significantly enriched patterns of gene expression following PHx within ERS UP, ERS DOWN and LIVER-ID genes - related to Fig.1.

Appendix Figure S3. Downregulation of LIVER-ID genes by acute ERS leading to partial hepatic dedifferentiation – related to Fig.1.

Appendix Figure S4. Additional characterization of H3K27ac DOWN regions - related to Fig.2.

Appendix Figure S5. Characterization of LIVER-ID TFs - related to Fig.2.

Appendix Figure S6. Additional characterization of LIVER-ID TFs and their global repression by acute ERS - related to Fig.2.

Appendix Figure S7. Additional characterization of NFIL3 induction and switch in the PAR-bZIP TF family expression pattern upon acute hepatic ERS - related to Fig.3.

Appendix Figure S8. Expression of PAR-bZIP TFs, LIVER-ID TFs and ERS UP genes in the liver of NFIL3 KO subjected to acute ERS - related to Fig.3.

Appendix Figure S9. ChIP-seq track visualization at additional genes related to xenobiotic metabolism whose maximal repression by acute ERS requires NFIL3 - related to Fig.3.

Appendix Figure S10. Interaction between the LIVER-ID TF FOXA2 and EP300 in MPH and BRD4 chromatin recruitment and levels in MPH subjected to acute ERS – related to Fig.4.

Appendix Figure S11. Detailed ERS UP and LIVER-ID gene expression data upon BRD4 inhibition - related to Fig.4.

Appendix Figure S12. LIVER-ID TF protein expression upon BRD4 inhibition - related to Fig.4.

Appendix Figure S13. Additional experiments defining a role for protein acetylation in the control of LIVER-ID gene expression by acute ERS - related to Fig.4.

Appendix Figure S14. Additional experiments defining a role for protein acetylation in the control of LIVER-ID gene expression by acute ERS - related to Fig.4.

Appendix Figure S15. Features of LIVER-ID genes overlapping BRD4 SE - related to Fig.4.

Appendix Figure S16. LIVER-ID TFs form an auto-/cross-binding transcriptional network - related to Fig.5.

Appendix Figure S17. Kinetics of LIVER-ID TF gene downregulation upon acute ERS in MPH - related to Fig.5.

Appendix Figure S18. Additional replicates showing the effect of MG132 or PP2 on ERS-induced LIVER-ID TF protein degradation - related to Fig.5.

Appendix Figure S19. ERS gene induction and loss of hepatic molecular identity in the sepsis^{BIM} model - related to Fig.6.

Appendix Figure S20. Analyses of the transcriptomic alterations in additional mouse liver injury models - related to Fig.6.

Appendix Figure S21. ERS gene induction and loss of LIVER-ID TFs in the sepsis^{BIM} model - related to Fig.6.

Appendix Figure S22. Expression switch of the PAR-bZIP TF family members in the liver of the sepsis^{BIM} and sepsis^{CLP} mouse models and additional data on LIVER-ID TF expression in the liver of sepsis^{BIM} mice pretreated with TUDCA - related to Fig.6-7.

Appendix Figure S23. Concomitant ERS gene induction and repression of LIVER-ID TF expression in the septic liver of deceased humans - related to Fig.7.

Appendix Figure S24. Effects of inhibitors of ERS sensors induced signaling on LIVER-ID gene repression and *Nfil3* induction - related to Discussion.

Appendix Figure S25. Additional controls verifying the specificity and relevance of our *in-vitro* acute ERS model in MPH - related to Discussion.

Appendix Figure S26. Loss of molecular identity upon acute ERS is also observed in skeletal muscle - related to Discussion.

Appendix Figure S27. Acute ERS in MPH does not trigger activation of CRMs associated with an inflammatory response - related to Discussion.

Appendix Figure S1

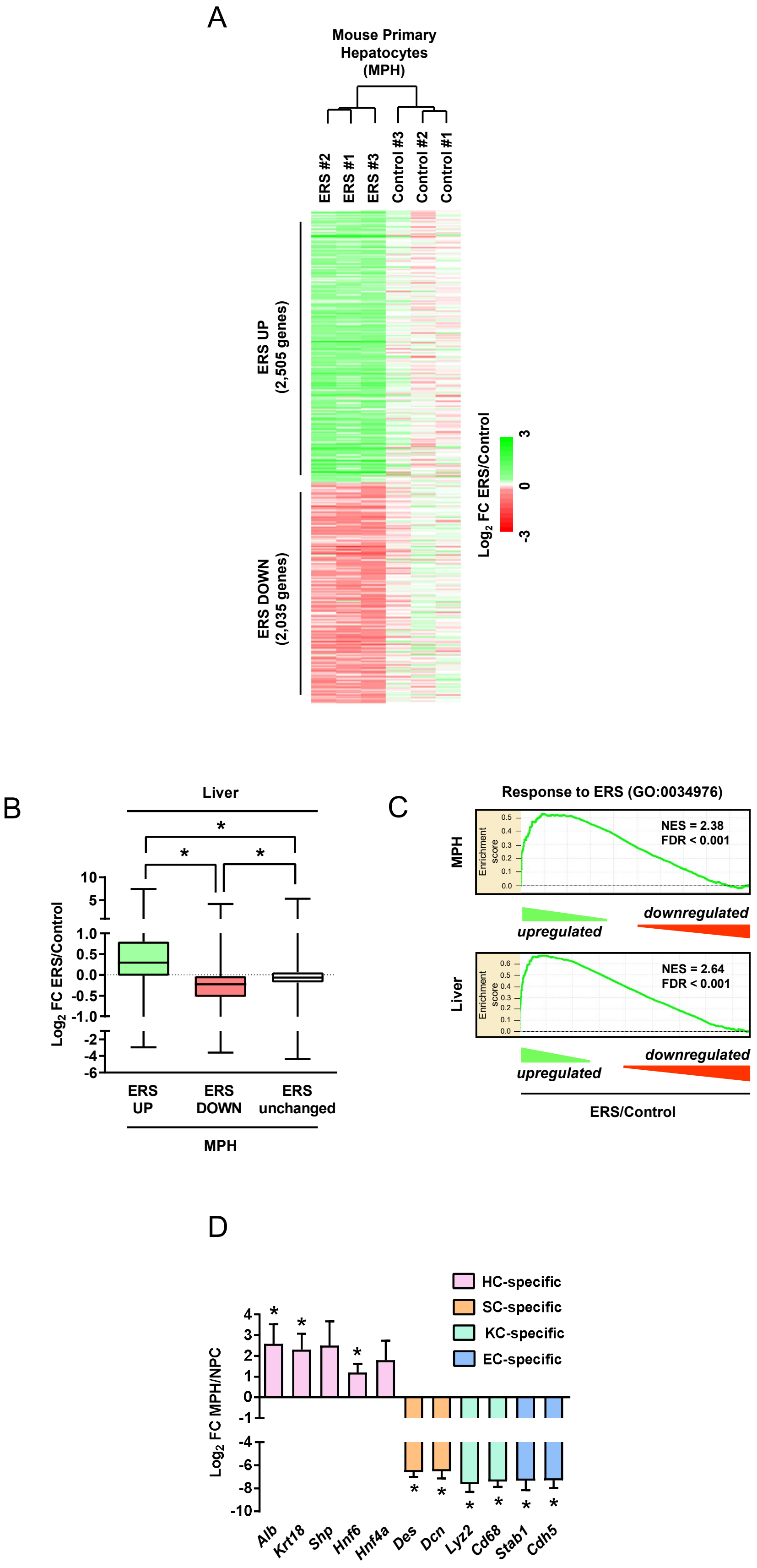

Appendix Figure S1. Additional characterization of transcriptomic changes induced by acute ERS - related to Fig.1. (A) Heatmap showing genes significantly modulated (FDR<0.05) by acute ERS in MPH. In all the figures from this study, ERS in MPH is defined as 4h treatment with 1µM thapsigargin unless indicated otherwise. Log_2 fold-change (FC) values from the microarray saturated at -3 for negative values <-3 and at 3 for positive values >3 are shown relative to the average expression among all Control (vehicle-treated) replicates. The hierarchical clustering tree is shown above the heatmap. **(B)** Box plots showing Log_2 FC for the genes from A in the liver of mice treated for 8h with 1µg/g body weight tunicamycin (ERS) vs vehicle (Control). These data were used again throughout the manuscript when referring to transcriptomic analyses of acute ERS in the mouse liver. One-way analysis of variance (ANOVA) with Welch's correction and Dunnett's Modified Tukey-Kramer pairwise multiple comparison test was used to assess statistical significance. **(C)** Enrichment plots from GSEA performed using the response to ERS gene set (GO:0034976) and transcriptomic changes induced by acute ERS in MPH (*upper panel*) or mouse liver (*lower panel*) as the ranked gene list. **(D)** Quality control of MPH preparation. RT-qPCR analyses of selected markers for hepatocytes (HC), stellate cells (SC), Kupffer cells (KC) and endothelial cells (EC) in mouse primary hepatocytes (MPH) and in the non-parenchymal cell (NPC) fraction from 3 independent preparations of MPH. Log_2 FC MPH/NPC are shown and one-sample t-test with Benjamini–Hochberg (BH) correction for multiple testing was used to determine if the mean is statistically different from 0.

Appendix Figure S2

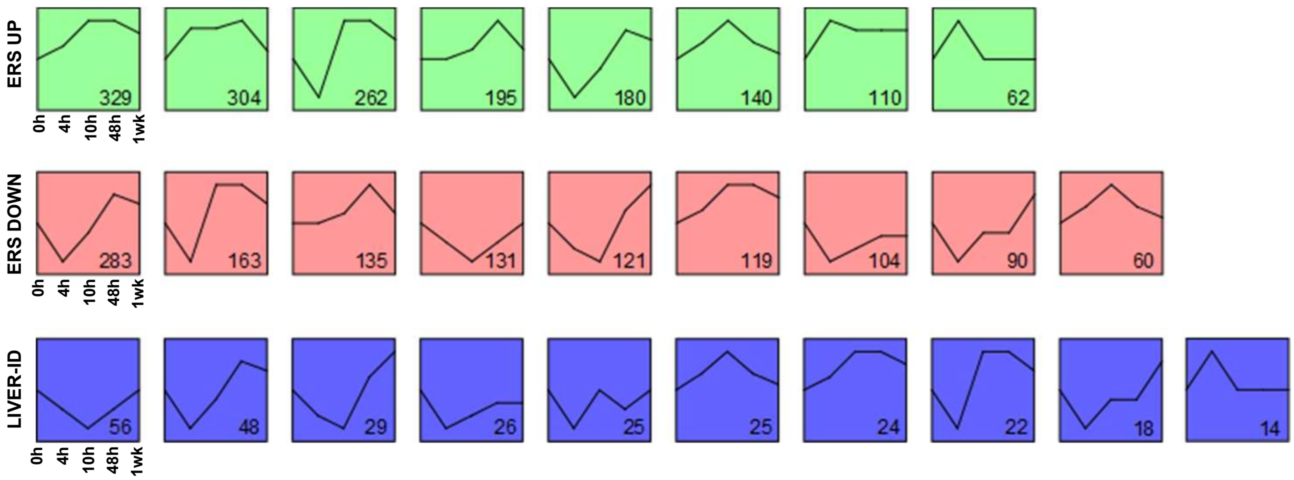

Appendix Figure S2. All model profiles with significantly enriched patterns of gene expression following PHx within ERS UP, ERS DOWN and LIVER-ID genes - related to Fig.1. Model profiles are organized relative to the number of genes they comprise (from left to right, gene numbers are indicated at the bottom of each box). Data show changes in expression at 4, 10, 48h and 1 week after PHx (0h) for genes comprised within each model profile of dynamic expression identified by STEM.

Appendix Figure S3

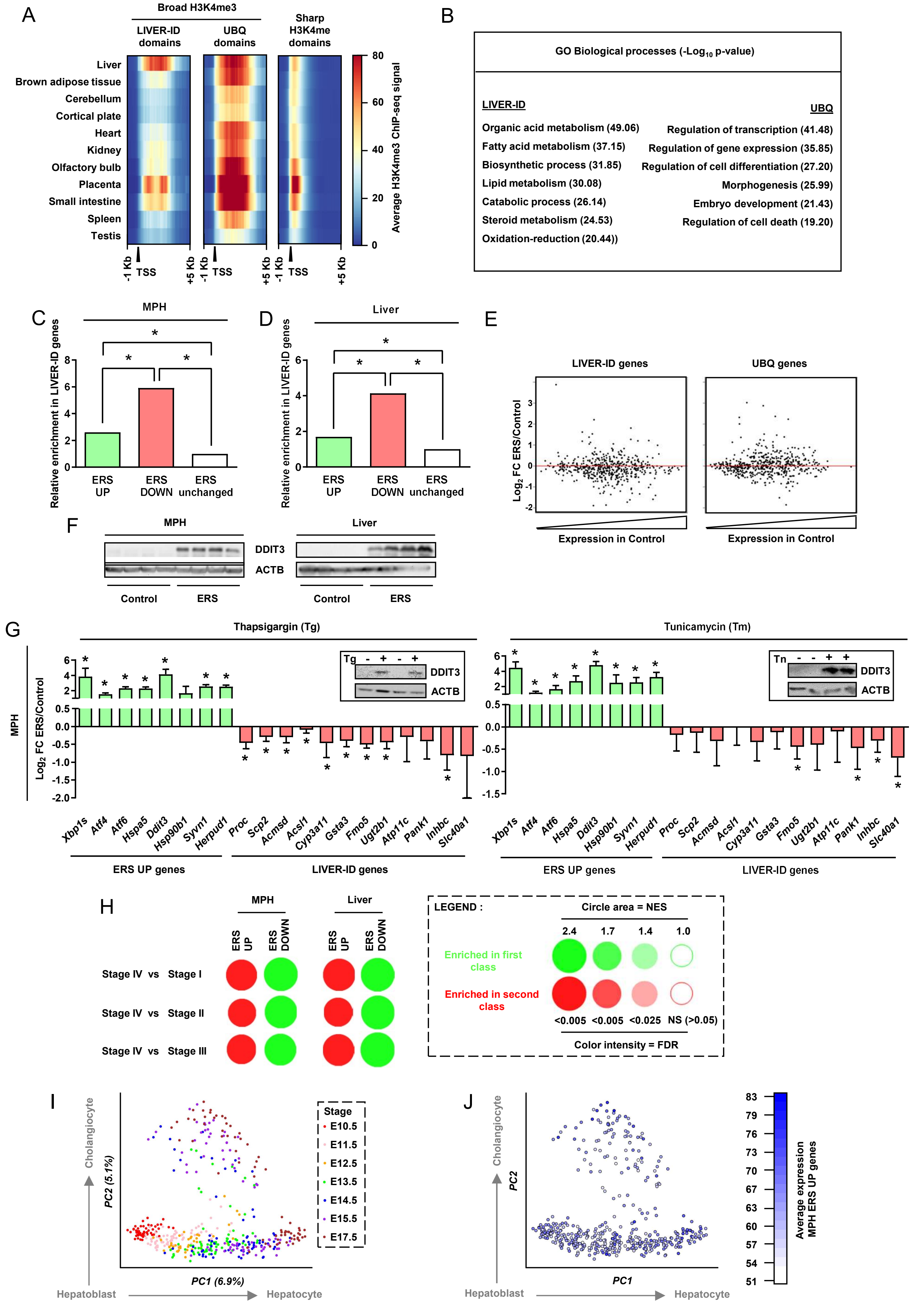

Appendix Figure S3. Downregulation of LIVER-ID genes by acute ERS leading to partial hepatic dedifferentiation – related to Fig.1. (A) Average H3K4me3 ChIP-seq signal around the transcription start site (TSS) of genes associated with broad H3K4me3 LIVER-ID domains, broad H3K4me3 UBQ domains or sharp H3K4me3 domains in mouse liver and 10 control tissues. The different domains were defined as described in Materials and Methods. **(B)** Functional enrichment analyses were performed using LIVER-ID and UBQ genes and the ToppGene Suite. GO Biological processes with Bonferronicorrected p-value $<10^{-6}$ were considered and similar terms were merged. Bonferroni-corrected pvalues ($-Log_{10}$) are shown. **(C-D)** The fraction of LIVER-ID genes among ERS UP, ERS DOWN or unchanged genes (i.e. genes not statistically affected by ERS) from MPH (C) or mouse liver (D) was defined and is displayed relative to that obtained for ERS unchanged genes arbitrarily set to 1. Chisquare test with BH correction for multiple testing was used to assess statistical significance. **(E)** Genes were positioned along the x-axis based on their basal expression levels in the non-treated MPH (Control) and their FC (Log_2) upon ERS was plotted along the y-axis. Note that the scale of the x-axis is similar between the 2 plots. These data show a lack of correlation between the extend of ERS-mediated gene repression and initial gene expression levels. **(F)** Total protein extracts from MPH (*left panel*) or mouse liver (*right panel*) were subjected to Western blot with an antibody against DDIT3. ACTB was used as loading control. **(G)** RT-qPCR analyses of selected ERS UP and LIVER-ID genes in MPH treated for 4h with vehicle (Control) or 1µM thapsigargin (*left panel ; reused from Fig.1L*) or 2µg/mL tunicamycin (*right panel*) (ERS) (3 to 9 independent experiments). One-sample t-test with BH correction for multiple testing was used to determine if the mean Log_2 FC ERS/Control is statistically different from 0. Total protein extracts from the same samples were subjected to Western blot (inset) with an antibody against DDIT3 to validate ERS response. ACTB was used as loading control. **(H)** Enrichment scores from GSEA performed using ERS UP or ERS DOWN genes as the gene set and transcriptomic changes occurring in the mouse liver at different developmental stages as the ranked gene lists were integrated and corrected for multiple testing using the BubbleGUM tool. **(I)** Principal component analysis (PCA) plot issued from the analysis of 447 hepatobiliary single-cell transcriptomes across seven developmental stages which are color-coded. Refer to Materials and Methods for a detailed description of the procedure. The hepatoblast-to-hepatocyte and hepatoblast-to-cholangiocyte differentiation paths are indicated with arrows. **(J)** Average expression of ERS UP genes from MPH in single-cells from the hepatobiliary lineage. See Materials and Methods together with Appendix Fig.S3I for details regarding data processing. The hepatoblast-to-hepatocyte and hepatoblast-to-cholangiocyte differentiation paths are indicated with arrows.

Appendix Figure S4

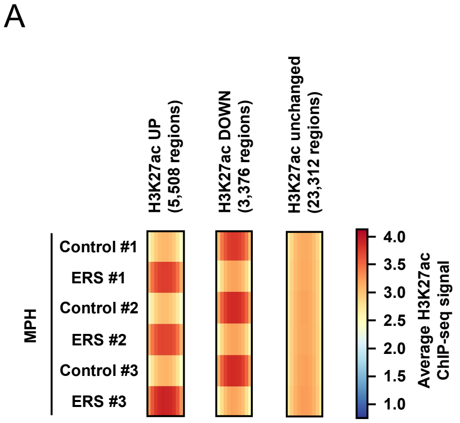

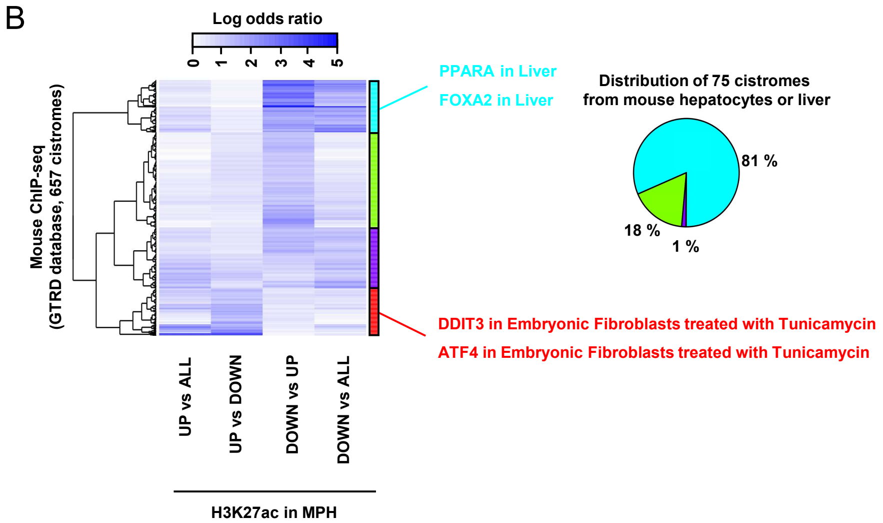

Appendix Figure S4. Additional characterization of H3K27ac DOWN regions - related to Fig.2. (A) Heatmaps showing the average H3K27ac ChIP-seq signals for regions with increased (H3K27ac UP), decreased (H3K27ac DOWN) or unchanged (H3K27ac unchanged) H3K27ac levels in MPH upon acute ERS for the 3 individual replicates. **(B)** Heatmap displaying results from LOLA where relative enrichment within H3K27ac regions (comparisons are indicated at the bottom) of TF binding sites issued from 657 cistromes (individual ChIP-seq datasets) was evaluated. ALL refers to the entire set of H3K27ac regions in MPH (i.e. H3K27ac UP, DOWN and unchanged). Hierarchical clustering trees are shown on the left side and 4 clusters of cistromes are highlighted on the right side of the heatmap. The red cluster identifies cistromes preferentially overlapping H3K27ac UP regions including DDIT3 and ATF4 cistromes from ERS samples. On the opposite, the blue cluster identifies cistromes preferentially overlapping H3K27ac DOWN regions including for example PPARA and FOXA2 cistromes from the mouse liver. In line, 81% (61 out of 75) of the cistromes obtained from mouse hepatocytes or mouse liver fall within this blue cluster indicating H3K27ac DOWN regions may represent CRMs densely co-bound by hepatic TFs. The list of the TFs within each cluster is provided in Table EV2.

Appendix Figure S5

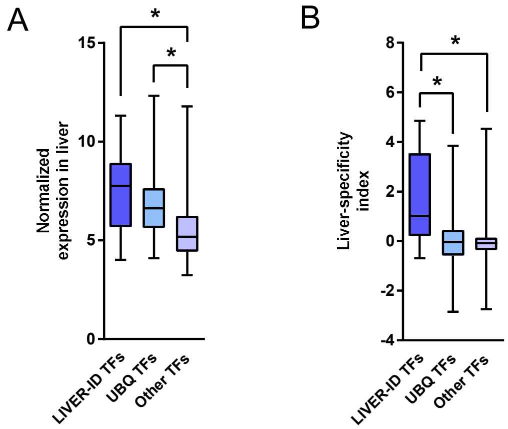

Appendix Figure S5. Characterization of LIVER-ID TFs - related to Fig.2. (A-B) Box plots showing normalized expression in mouse liver (A) and liver-specificity index (B) for LIVER-ID TFs, UBQ TFs and other TFs. One-way ANOVA with Welch's correction and Dunnett's Modified Tukey-Kramer pairwise multiple comparison test was used to assess statistical significance.

Appendix Figure S6

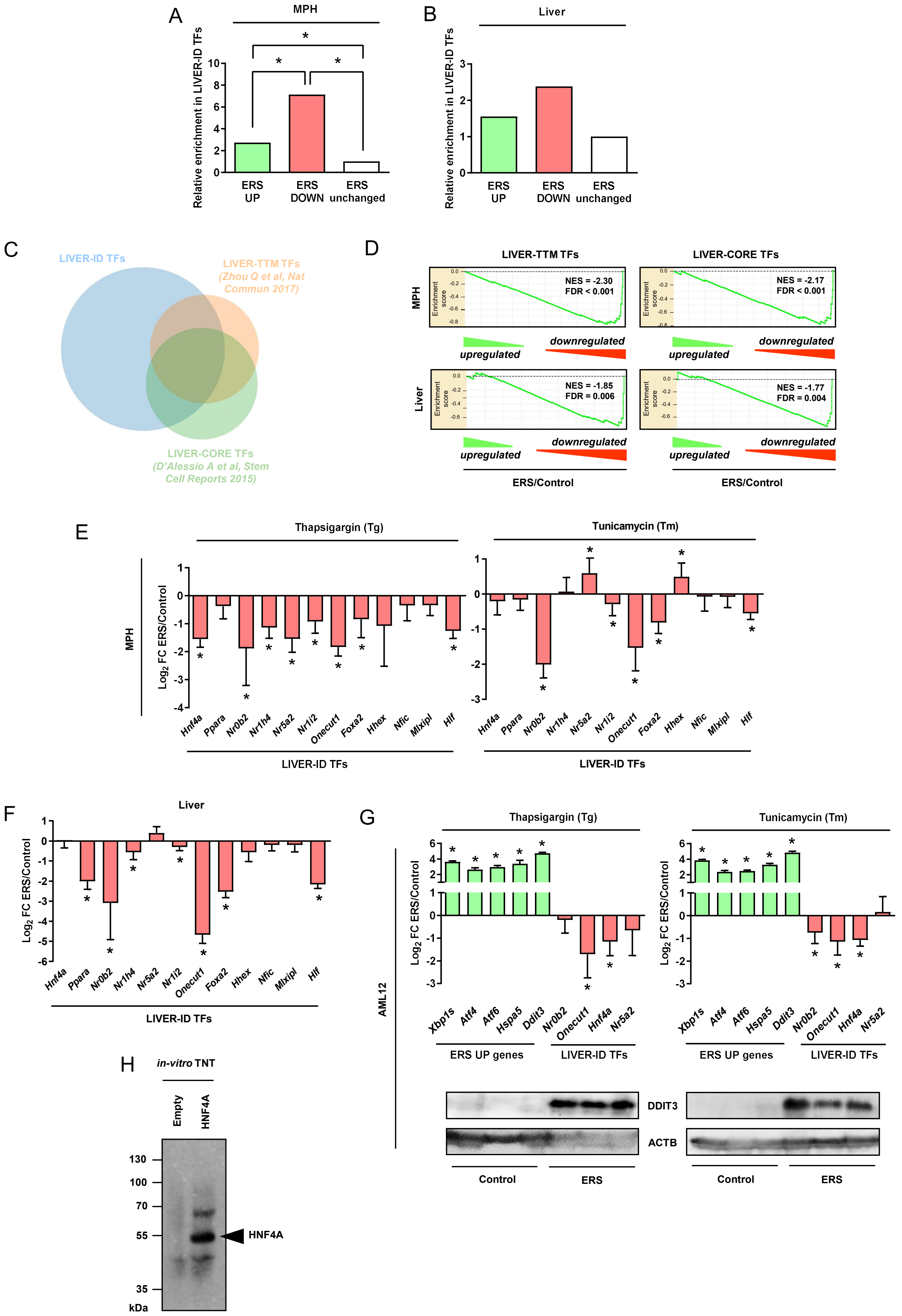

Appendix Figure S6. Additional characterization of LIVER-ID TFs and their global repression by acute ERS - related to Fig.2. (A-B) The fraction of LIVER-ID TFs among ERS UP, ERS DOWN or unchanged genes (i.e. genes not statistically affected by ERS) from MPH (A) or mouse liver (B) was defined and is displayed relative to that obtained for ERS unchanged genes arbitrarily set to 1. Fisher's exact test with BH correction for multiple testing was used to assess statistical significance. **(C)** Venn diagram showing the overlap between the 43 LIVER-ID TFs listed in Table EV1, the 19 LIVER-TTM TFs from (Zhou et al., 2017) and the 20 top ranked LIVER-CORE TFs from (D'Alessio et al., 2015). **(D)** Enrichment plots from GSEA performed using LIVER-TTM TFs (*left panels*) or LIVER-CORE TFs (*right panels*) from C as the gene set and transcriptomic changes induced by acute ERS in MPH (*upper panels*) or mouse liver (*lower panels*) as the ranked gene list. **(E)** RT-qPCR analyses of selected LIVER-ID TFs in MPH treated for 4h with vehicle (Control) or 1µM thapsigargin (*left panel ; reused from Fig.2F*) or 2µg/mL tunicamycin (*right panel*) (ERS) (4 to 7 independent experiments). One-sample t-test with BH correction for multiple testing was used to determine if the mean Log_2 FC ERS/Control is statistically different from 0. **(F)** RT-qPCR analyses of selected LIVER-ID TFs monitoring expression changes induced by acute ERS in mouse liver (5 mice per group). One-sample t-test with BH correction for multiple testing was used to determine if the mean Log_2 FC ERS/Control is statistically different from 0. **(G)** *Upper panels*, RT-qPCR analyses of selected ERS UP genes and LIVER-ID TFs in AML12 cells treated for 4h with vehicle (Control) or 1µM thapsigargin (*left panel*) or 2µg/mL tunicamycin (*right panel*) (ERS) (3 to 6 independent experiments). One-sample t-test with BH correction for multiple testing was used to determine if the mean Log_2 FC ERS/Control is statistically different from 0. *Lower panels*, Total protein extracts from the same samples were subjected to Western blot with an antibody against DDIT3. ACTB was used as loading control. **(H)** Quality control of the HNF4A antibody. Proteins issued from *in-vitro* transcription and translation (*invitro* TNT) of empty or HNF4A expressing plasmids were subjected to Western blot with an antibody against HNF4A.

Appendix Figure S7

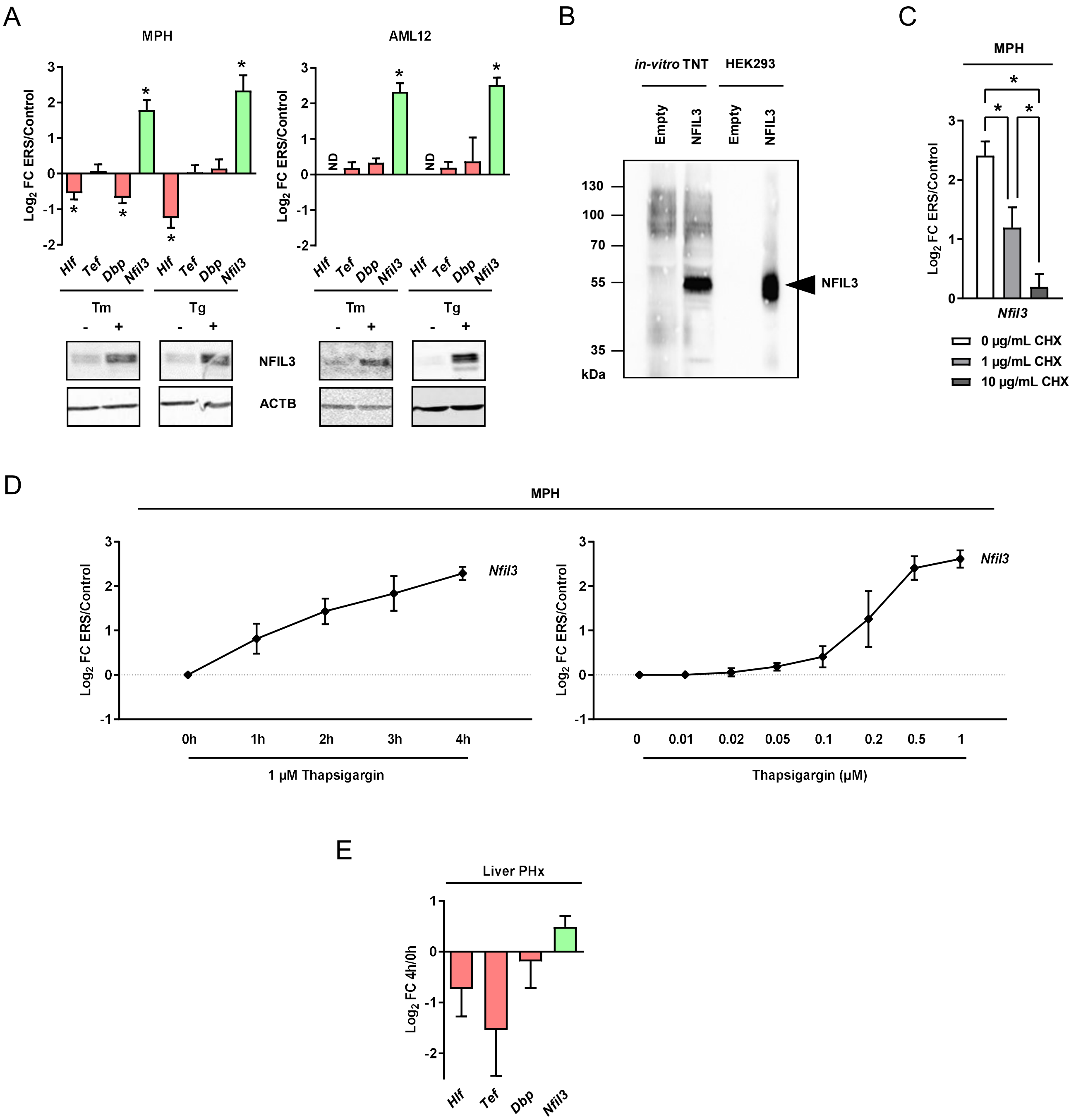

Appendix Figure S7. Additional characterization of NFIL3 induction and switch in the PAR-bZIP TF family expression pattern upon acute hepatic ERS - related to Fig.3. (A) *Upper panels,* RT-qPCR analyses of *Hlf*, *Tef*, *Dbp* and *Nfil3* expression in MPH (4 independent experiments) (*left*) and AML12 (3 independent experiments) (*right*) treated for 4h with 2µg/mL tunicamycin (Tunica) or 1µM thapsigargin (Thapsi). One-sample t-test with BH correction for multiple testing was used to determine if the mean Log_2 FC ERS/Control is statistically different from 0. ND = not detected. *Lower panels,* Total protein extracts from the same samples were subjected to Western blot with an antibody against NFIL3. ACTB was used as loading control. **(B)** Quality control of the NFIL3 antibody. Proteins issued from *in-vitro* transcription and translation (*in-vitro* TNT) of empty or NFIL3 expressing plasmids (*left*) or total protein extracts from HEK293 cells transfected with the same plasmids (*right*) were subjected to Western blot with an antibody against NFIL3. **(C)** RT-qPCR analyses of *Nfil3* expression in MPH co-treated for 4h with 0, 1 or 10 µg/mL cycloheximide (CHX) and 1µM thapsigargin (ERS) (3 independent experiments). Mean Log_2 FC ERS/Control are shown for every CHX dose. One-way ANOVA with Bonferroni's post-hoc test was used to assess statistical significance. **(D)** RT-qPCR analyses of *Nfil3* expression in MPH treated with 1µM thapsigargin for 0, 1, 2, 3 or 4h (4 independent experiments) (*left panel*) or for 4h with increasing thapsigargin doses as indicated (3 independent experiments) (*right panel*). **(E)** Shown are Log_2 FC of the PAR-bZIP TF family members issued from the transcriptomic analyses following mouse liver PHx (4h) relative to their basal expression levels.

Appendix Figure S8

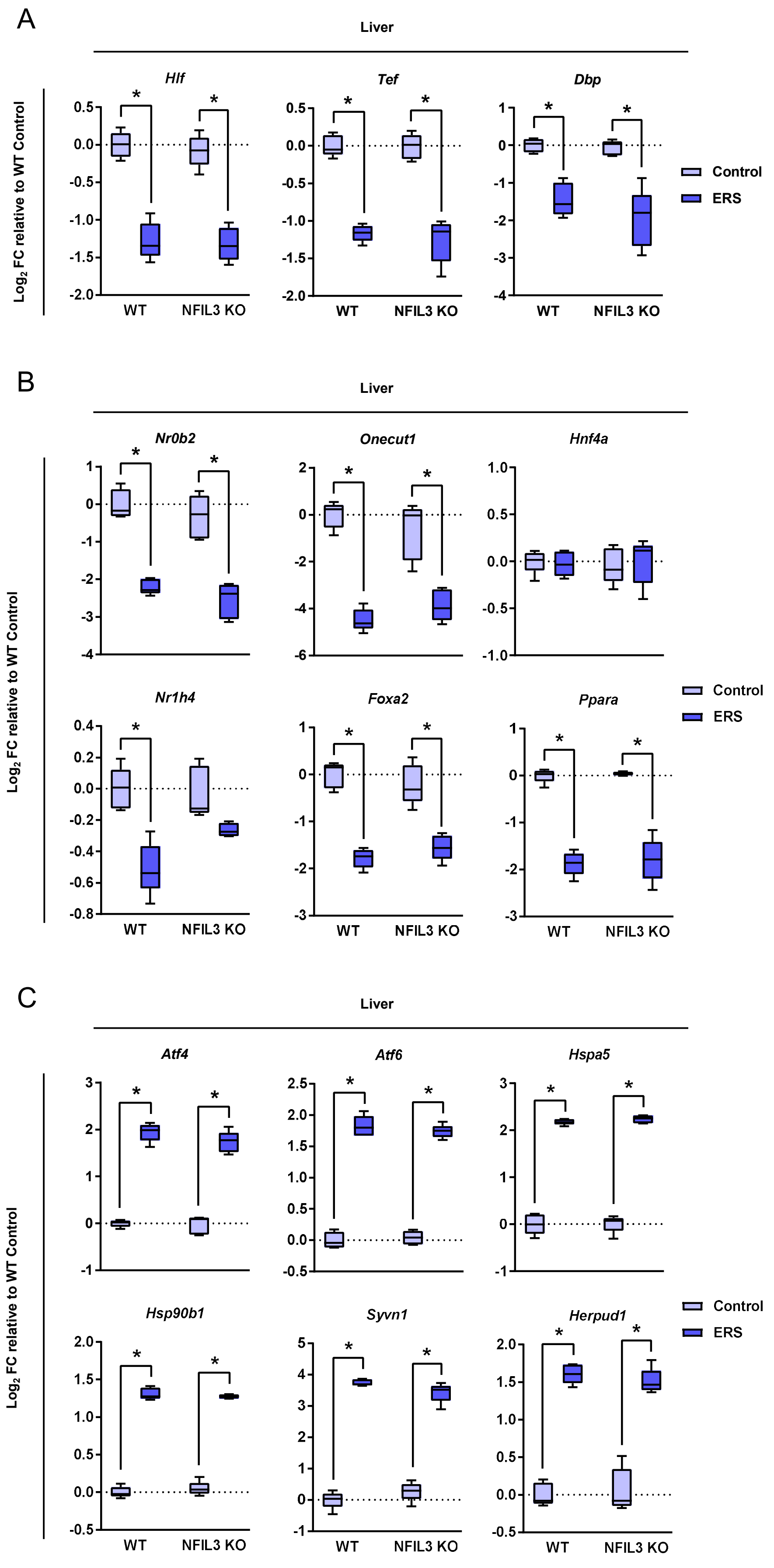

Appendix Figure S8. Expression of PAR-bZIP TFs, LIVER-ID TFs and ERS UP genes in the liver of NFIL3 KO subjected to acute ERS - related to Fig.3. Box plots showing mRNA expression for the indicated PAR-bZIP TFs **(A)**, LIVER-ID TFs **(B)** and ERS UP genes **(C)** issued from the transcriptomic analyses of the liver of NFIL3 KO or WT littermate mice subjected to acute ERS. Shown are Log_2 FC relative to the mean normalized expression in the WT Control group (5 mice per group). Two-way ANOVA with Bonferroni's post-hoc test was used to assess statistical significance.

Appendix Figure S9

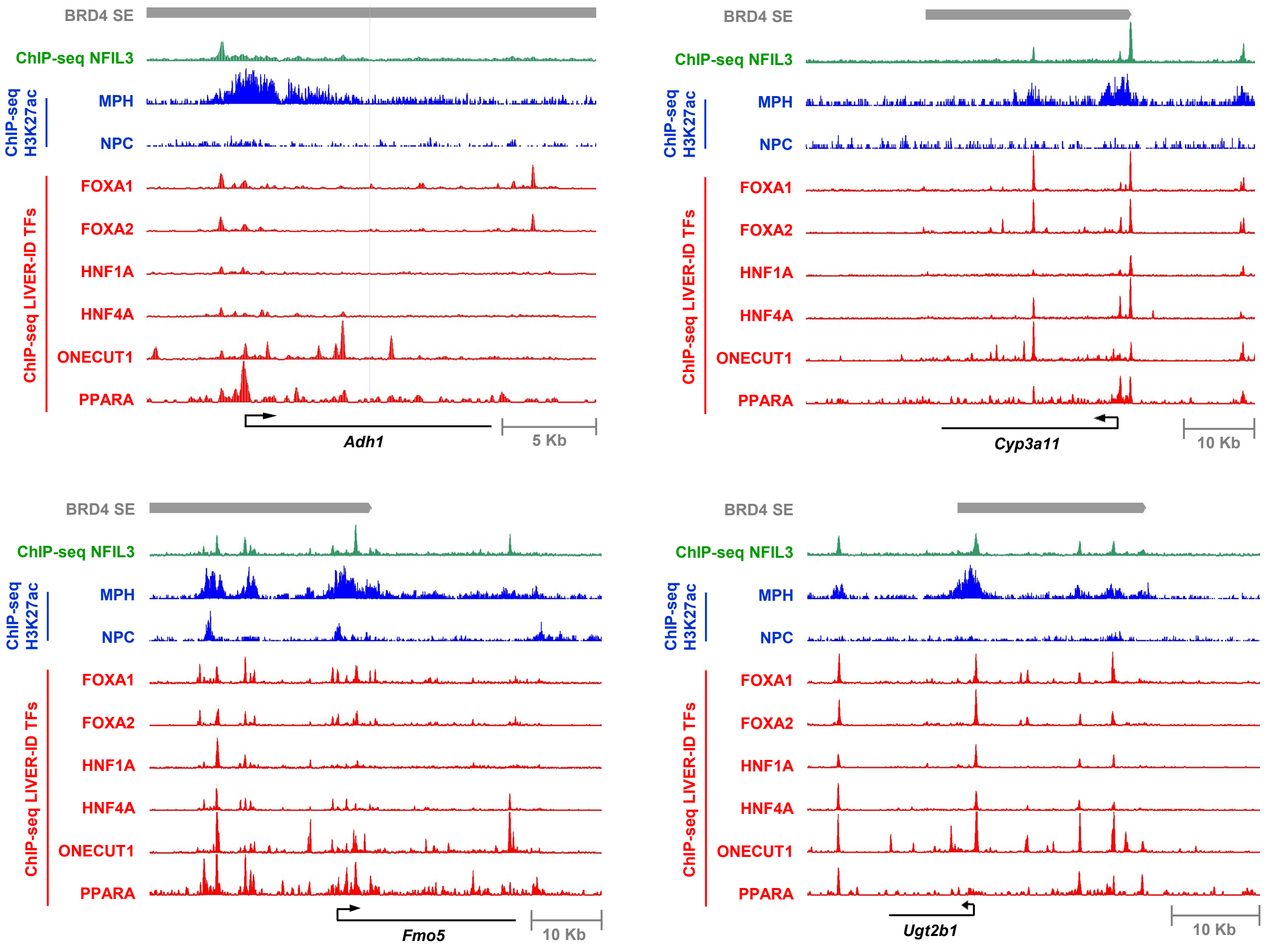

Appendix Figure S9. ChIP-seq track visualization at additional genes related to xenobiotic metabolism whose maximal repression by acute ERS requires NFIL3 - related to Fig.3. The Integrated Genome Browser (IGB) was used to visualize ChIP-seq profiles for NFIL3 (green) and several LIVER-ID TFs (red) in the mouse liver at the *Adh1*, *Cyp3a11*, *Fmo5* and *Ugt2b1* gene loci. Levels of H3K27ac in MPH and cells from the non-parenchymal fraction (NPC) are shown in blue. The grey bars indicate the position of BRD4 SE.

Appendix Figure S10

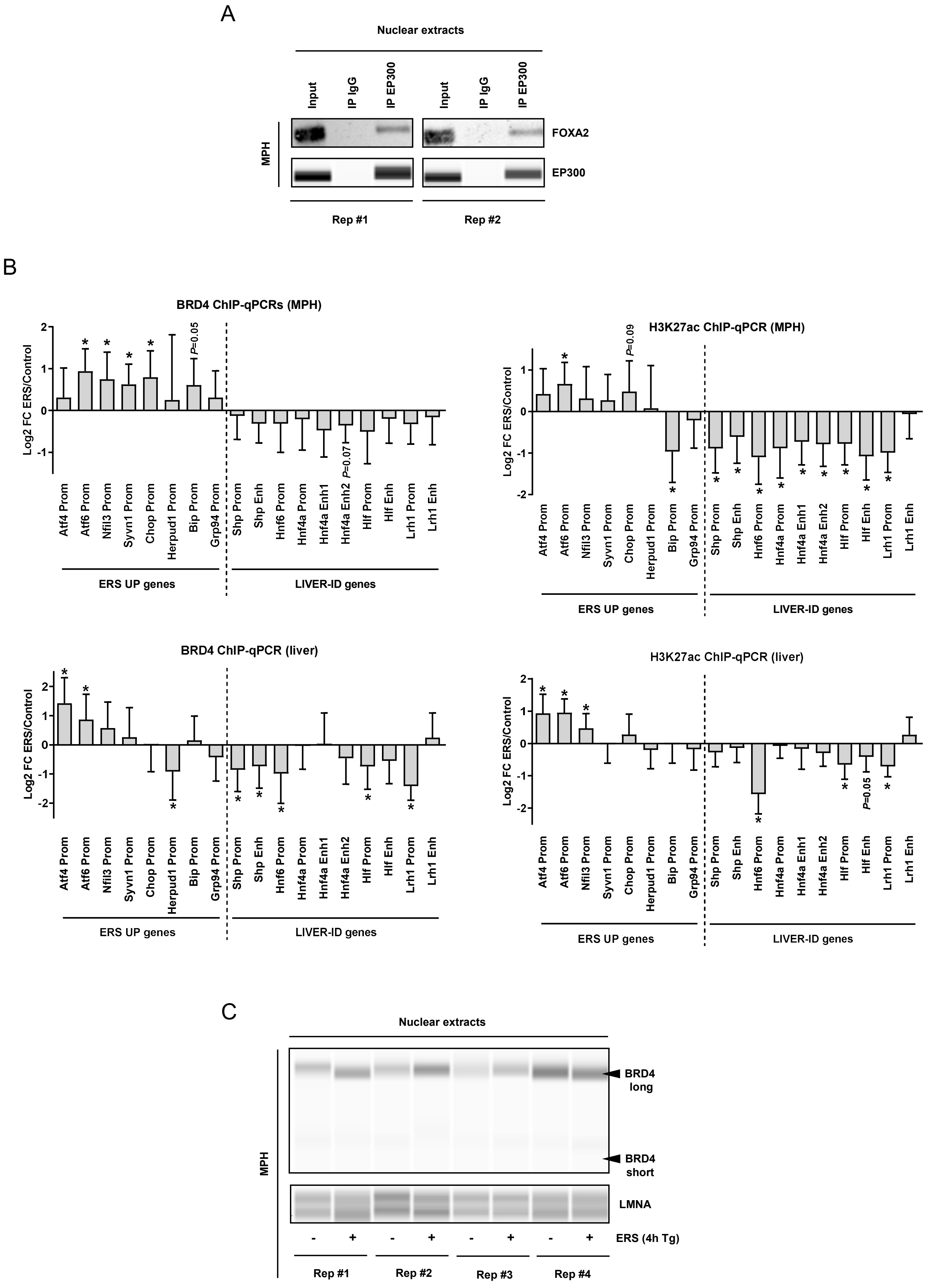

Appendix Figure S10. Interaction between the LIVER-ID TF FOXA2 and EP300 in MPH and BRD4 chromatin recruitment and levels in MPH subjected to acute ERS – related to Fig.4. **(A)** Nuclear extracts from MPH were subjected to immunoprecipitation with an antibody against EP300. Beadbound material was analysed by Western blot with antibodies against FOXA2 or EP300. Results obtained from 2 independent biological replicates are shown. **(B)** Related to Fig.4A. BRD4 occupancy and H3K27ac levels at individual regulatory regions associated with ERS UP or LIVER-ID genes (listed in Table EV5) were assessed by ChIP-qPCR in MPH (10 independent experiments) or mouse liver (10 mice per group) to define changes induced by acute ERS. Prom; promoter. Enh, potential enhancer. Onesample t-test with BH correction for multiple testing was used to determine if the mean Log_2 FC ERS/Control is statistically different from 0. **(C)** Simple Western immunoassays showing Additional biological replicates independent from the one displayed in Fig.4B used to monitor BRD4 protein levels in MPH subjected to ERS.

Appendix Figure S11

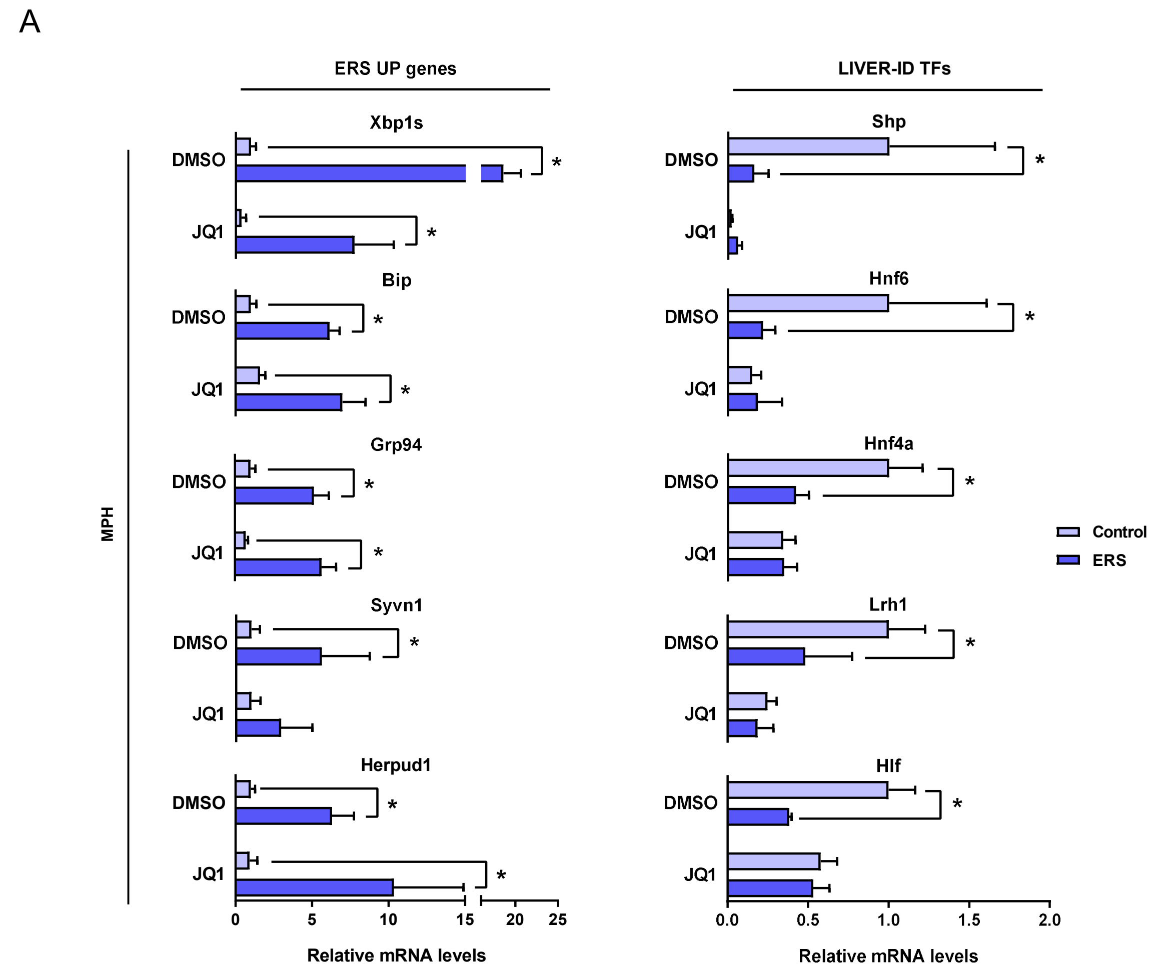

Appendix Figure S11 (continued)

B

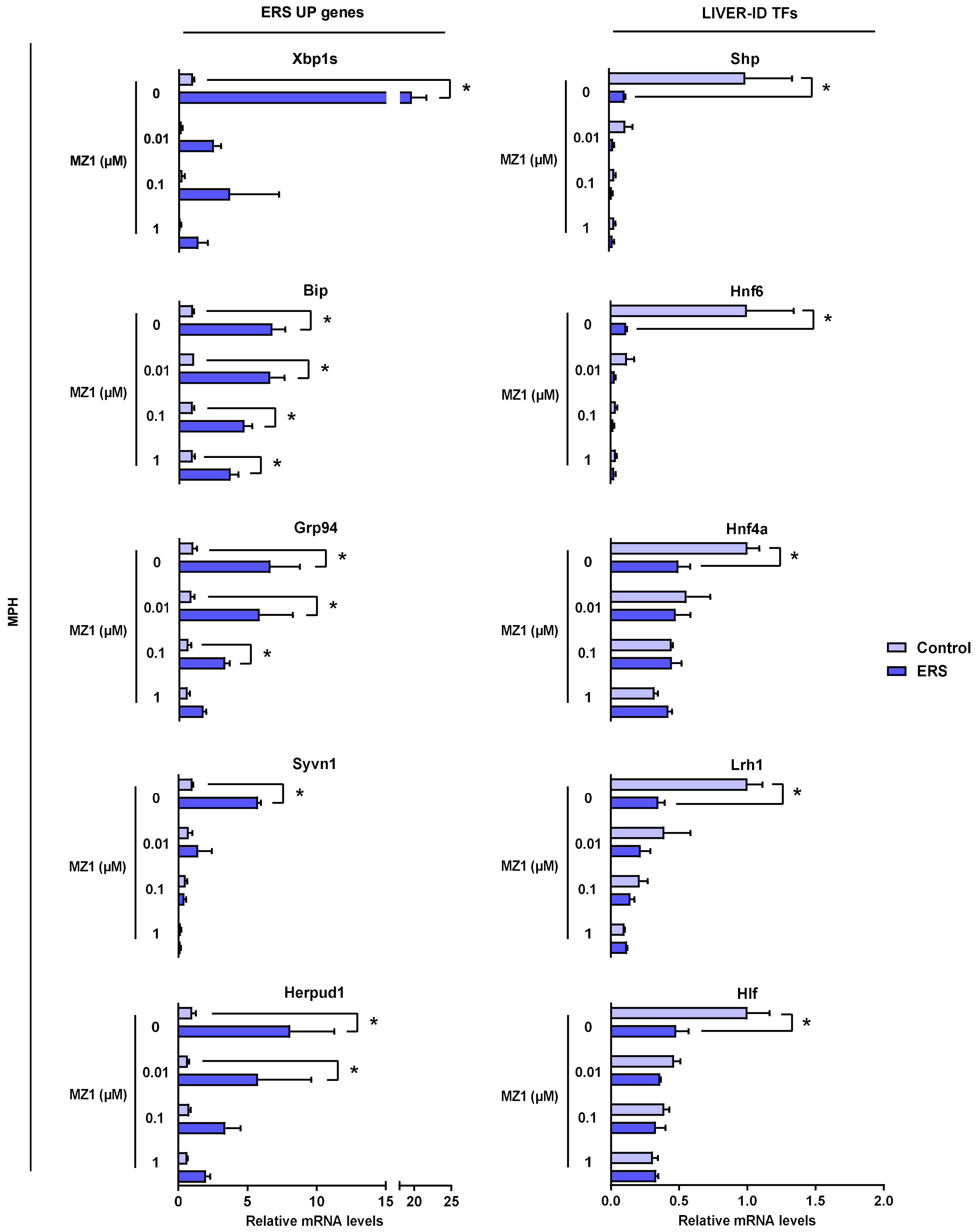

Appendix Figure S11. Detailed ERS UP and LIVER-ID gene expression data upon BRD4 inhibition - related to Fig.4. RT-qPCR analyses showing relative mRNA levels of selected ERS UP and LIVER-ID genes in MPH pre-treated with 500nM JQ1 **(A)** or increasing MZ1 doses as indicated **(B)** followed by addition of 1µM thapsigargin (ERS) for 4h (3 independent experiments). Two-way ANOVA with Bonferroni's post-hoc test was used to assess statistical significance.

Appendix Figure S12

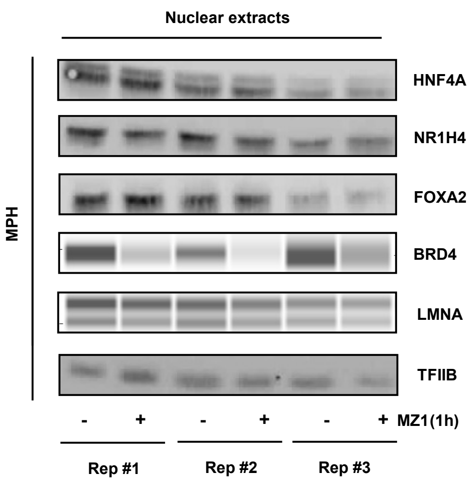

Appendix Figure S12. LIVER-ID TF protein expression upon BRD4 inhibition - related to Fig.4. Nuclear extracts from MPH treated with 0.01µM MZ1 for 1h were subjected to Western blot or Simple Western immunoassay with antibodies against HNF4A, NR1H4, FOXA2 or BRD4. LMNA and TFIIB were used as loading controls.

Appendix Figure S13

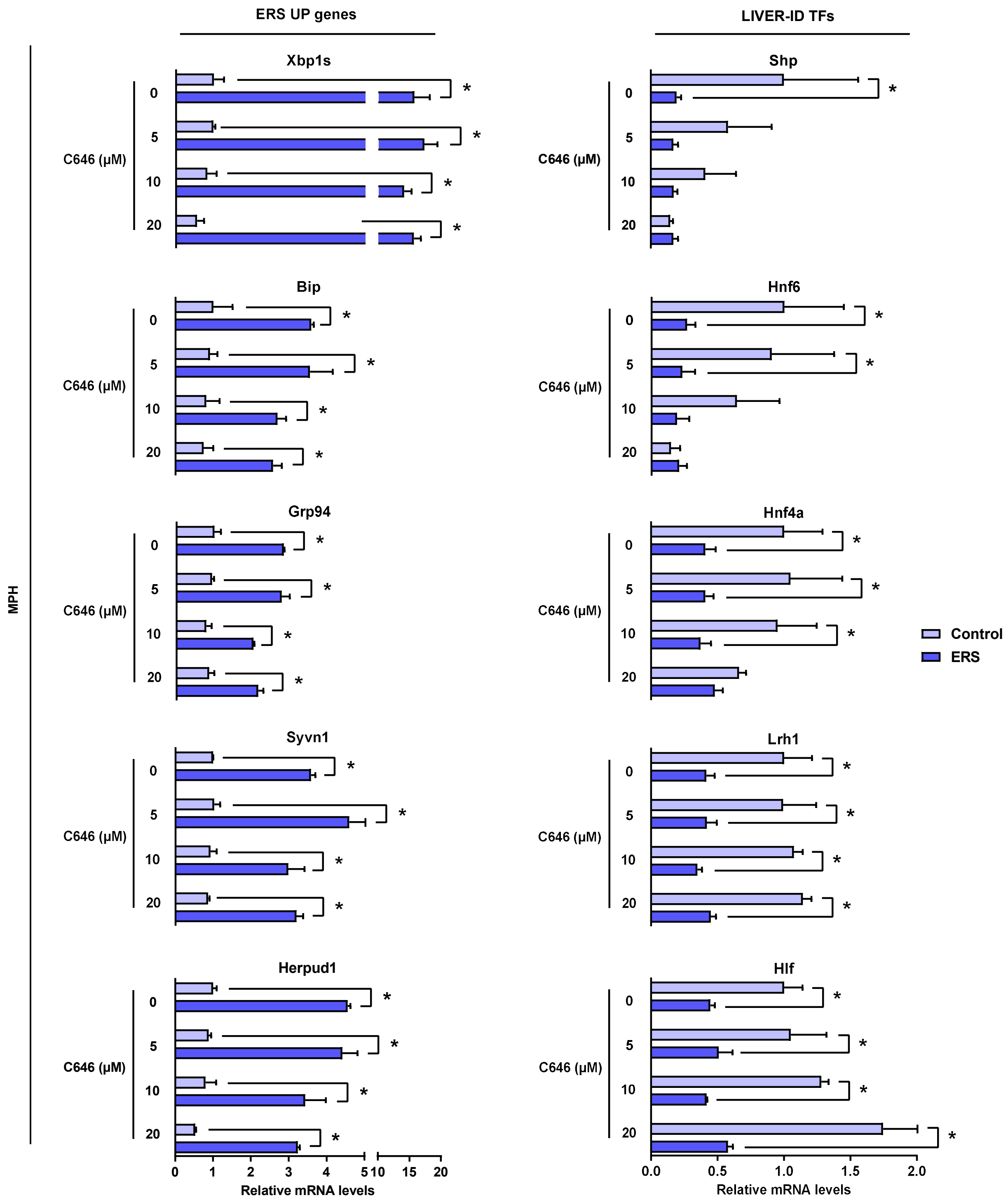

Appendix Figure S13. Additional experiments defining a role for protein acetylation in the control of LIVER-ID gene expression by acute ERS - related to Fig.4. RT-qPCR analyses showing relative mRNA levels of selected ERS UP and LIVER-ID genes in MPH co-treated for 4h with increasing C646 doses as indicated and 1µM thapsigargin (ERS) (3 independent experiments). Two-way ANOVA with Bonferroni's post-hoc test was used to assess statistical significance.

Supplemental Figure S14

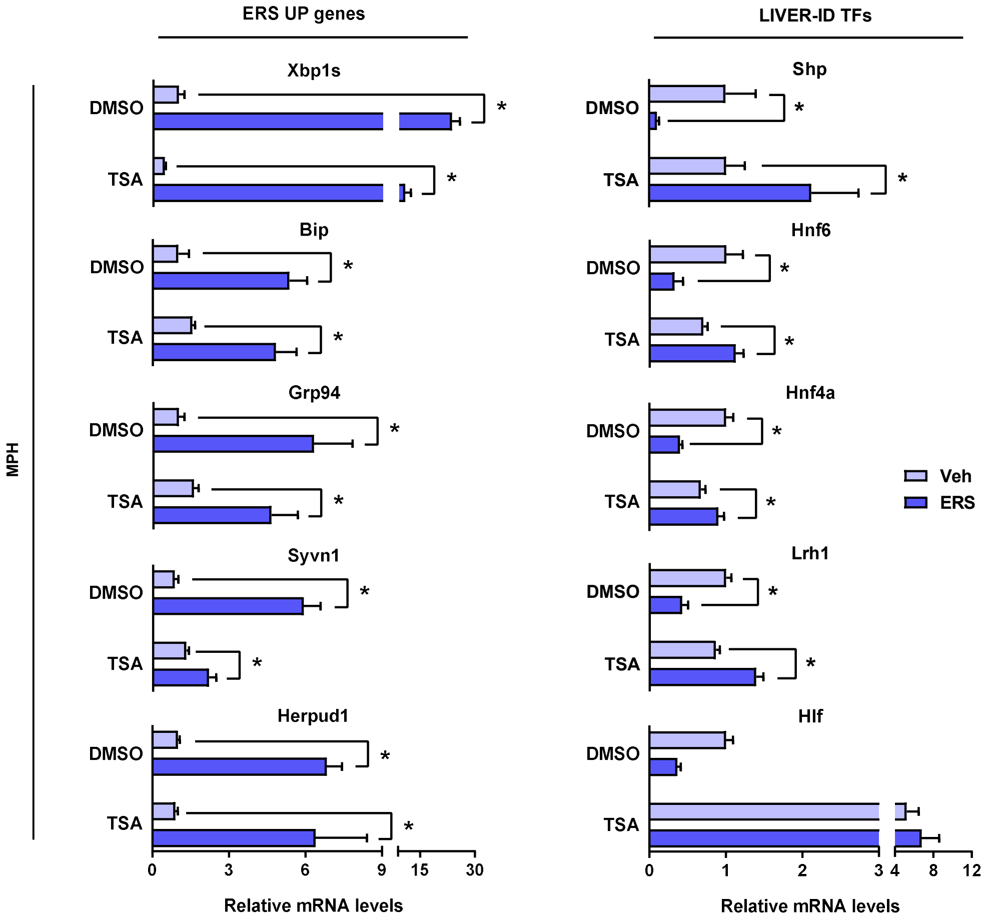

Appendix Figure S14. Additional experiments defining a role for protein acetylation in the control of LIVER-ID gene expression by acute ERS - related to Fig.4. RT-qPCR analyses showing relative mRNA levels of selected ERS UP and LIVER-ID genes in MPH co-treated for 4h with 1µM trichostatin A (TSA) and 1µM thapsigargin (ERS) (3 independent experiments). Two-way ANOVA with Bonferroni's post-hoc test was used to assess statistical significance.

Appendix Figure S15

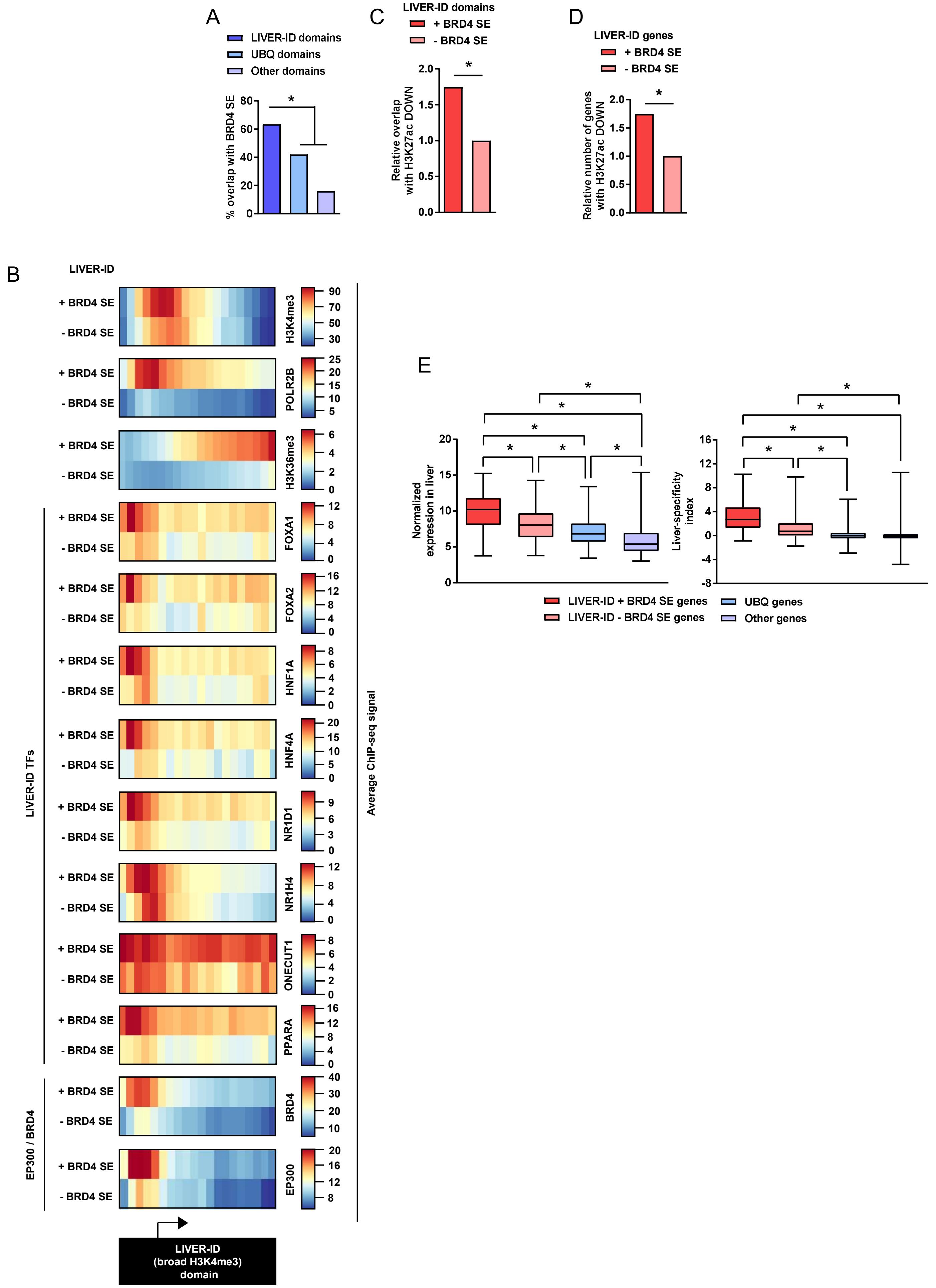

Appendix Figure S15. Features of LIVER-ID genes overlapping BRD4 SE - related to Fig.4. (A) The percentage of LIVER-ID domains, UBQ domains and other domains overlapping BRD4 SE is indicated. Fisher's exact test with BH correction for multiple testing was used to assess statistical significance. **(B)** Heatmaps showing average ChIP-seq signals in mouse liver for H3K4me3, POL2RB, H3K36me3, several LIVER-ID TFs as well as BRD4 and EP300 at LIVER-ID domains overlapping (+) or not (-) with BRD4 SE. **(C)** The number of LIVER-ID + BRD4 SE domains overlapping H3K27ac DOWN regions is indicated relative to the number of LIVER-ID - BRD4 SE domains overlapping H3K27ac DOWN regions. Fisher's exact test was used to assess statistical significance. **(D)** The number of LIVER-ID + BRD4 SE genes common with H3K27ac DOWN assigned genes is indicated relative to the number of LIVER-ID - BRD4 SE genes common with H3K27ac DOWN assigned genes. Fisher's exact test was used to assess statistical significance. **(E)** Box plots showing normalized expression in mouse liver (*left panel*) and liver-specificity index (*right panel*) for LIVER-ID + BRD4 SE genes, LIVER-ID - BRD4 SE genes, UBQ genes and other genes. One-way ANOVA with Welch's correction and Dunnett's Modified Tukey-Kramer pairwise multiple comparison test was used to assess statistical significance.

Appendix Figure S16

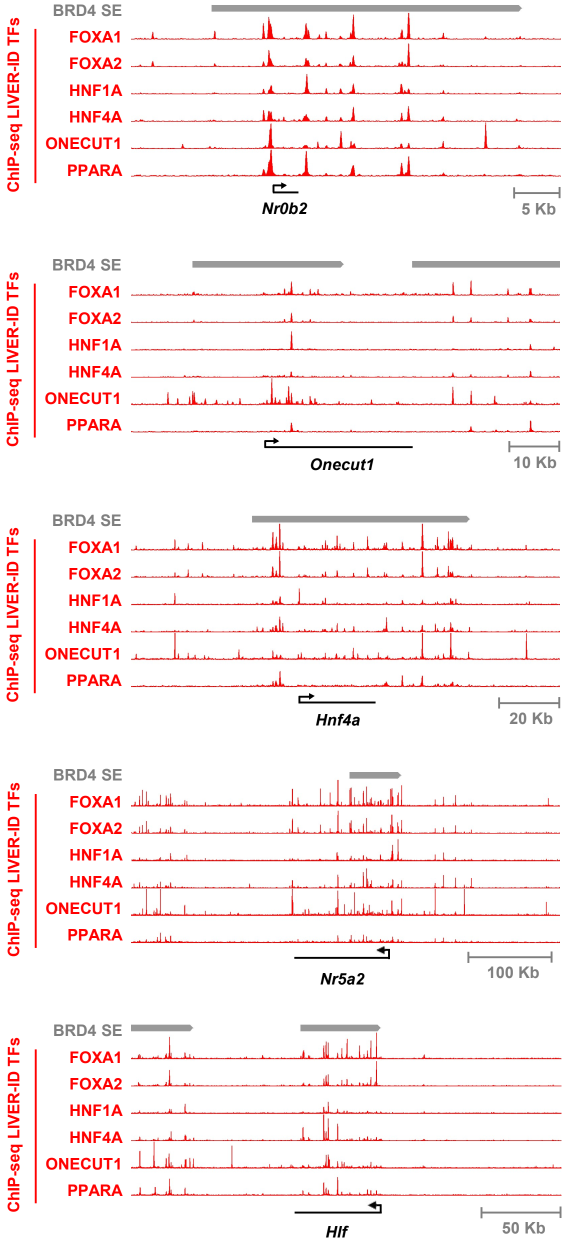

Appendix Figure S16. LIVER-ID TFs form an auto-/cross-binding transcriptional network - related to Fig5. The Integrated Genome Browser (IGB) was used to visualize ChIP-seq profiles for several LIVER-ID TFs (red) in the mouse liver at the indicated loci. The grey bars indicate the position of BRD4 SE.

Appendix Figure S17

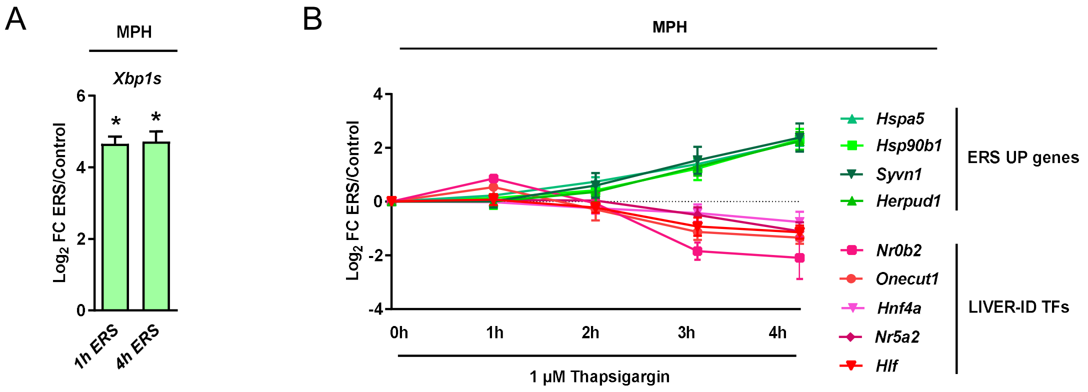

Appendix Figure S17. Kinetics of LIVER-ID TF gene downregulation upon acute ERS in MPH - related to Fig.5. (A) RT-qPCR analyses of *Xbp1s* expression in MPH treated with vehicle (Control) or 1µM thapsigargin (ERS) for 1 or 4h (4 independent experiments). One-sample t-test with BH correction for multiple testing was used to determine if the mean Log_2 FC ERS/Control is statistically different from 0**. (B)** RT-qPCR analyses of selected ERS UP genes and LIVER-ID TFs in MPH treated with vehicle (Control) or 1µM thapsigargin (ERS) for 0, 1, 2, 3 or 4h (4 independent experiments).

Appendix Figure S18

A

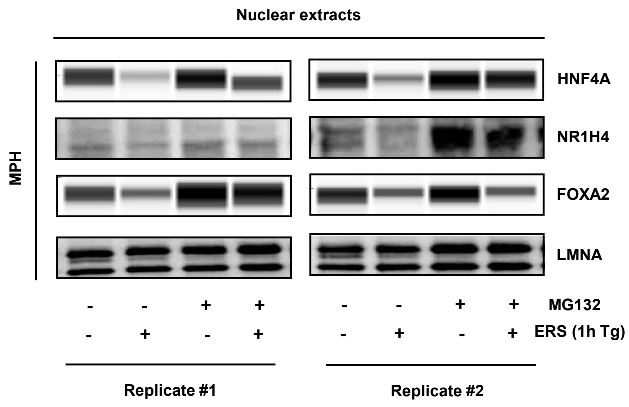

B

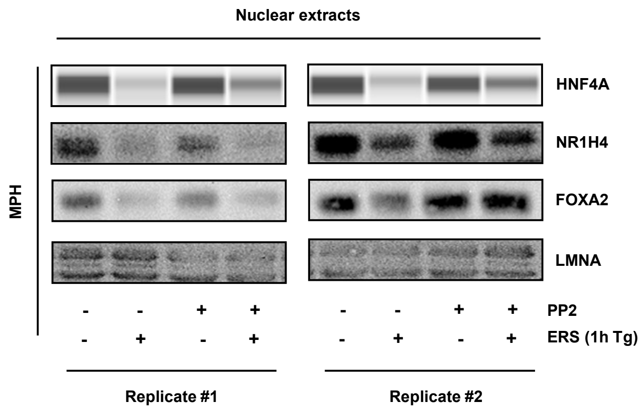

Appendix Figure S18. Additional replicates showing the effect of MG132 or PP2 on ERS-induced LIVER-ID TF protein degradation - related to Fig.5. Nuclear extracts from MPH pre-treated for 30min with 10µM MG132 **(A)** or 10µM PP2 **(B)** followed by addition of 1µM thapsigargin (ERS) for 1h were subjected to Western blot or Simple Western immunoassay with antibodies against HNF4A, NR1H4 or FOXA2. LMNA was used as loading control. Additional biological replicates independent from those shown in Fig.5F are displayed in each panel.

Appendix Figure S19

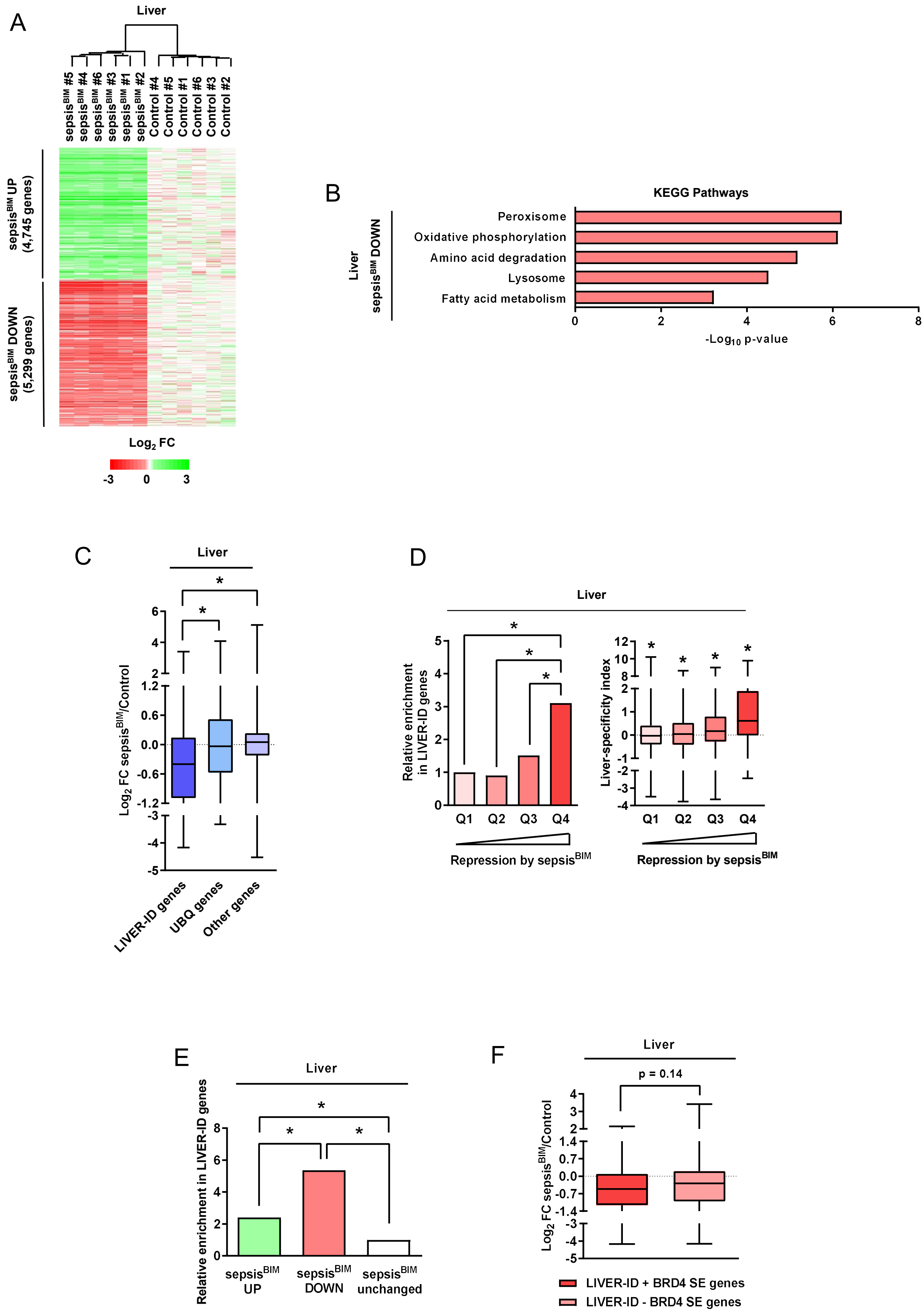

Appendix Figure S19. ERS gene induction and loss of hepatic molecular identity in the sepsis^{BIM} model - related to Fig.6. (A) Heatmap showing genes significantly modulated (FDR<0.05) in the liver of sepsis^{BIM} mice 16h after injection of live *E. coli* (6 mice per group). Log_2 FC values from the microarray saturated at -3 for negative values <-3 and at 3 for positive values >3 are shown relative to the average expression among all Control replicates. The hierarchical clustering tree is shown above the heatmap. **(B)** Functional enrichment analyses were performed using sepsis^{BIM} DOWN genes from A and the ToppGene Suite. KEGG Pathways with Bonferroni-corrected p-value <10^{-3} were considered and similar terms were merged. Bonferroni-corrected p-values (-Log_{10}) are shown. **(C)** Box plots showing Log_2 FC sepsis^{BIM} vs Control mouse liver for LIVER-ID genes, UBQ genes and other genes. One-way ANOVA with Welch's correction and Dunnett's Modified Tukey-Kramer pairwise multiple comparison test was used to assess statistical significance. **(D)** Genes repressed in sepsis^{BIM} mouse liver were ranked based on their Log_2 FC sepsis^{BIM} vs Control and divided into quartiles (increased repression from Q1 to Q4). *Left panel* The fraction of LIVER-ID genes in the 4 quartiles was defined and is displayed relative to that obtained for Q1 arbitrarily set to 1. Chi-square test with BH correction for multiple testing was used to assess statistical significance. *Right panel* Liver-specificity index was calculated for the genes of each quartile and is reported as in Fig.1E. One-sample t-test with BH correction for multiple testing was used to determine if the index is statistically different from 0. **(E)** The fraction of LIVER-ID genes among the upregulated and downregulated genes in sepsis^{BIM} vs Control mouse liver is indicated relative to the fraction of LIVER-ID genes among the genes not statistically significantly affected by sepsis^{BIM} (unchanged) arbitrarily set to 1. Chi-square test with BH correction for multiple testing was used to assess statistical significance. **(F)** Box plots showing Log_2 FC sepsis^{BIM} vs Control mouse liver for LIVER-ID + BRD4 SE and LIVER-ID - BRD4 SE genes. Student's t-test was used to assess statistical significance.

Appendix Figure S20

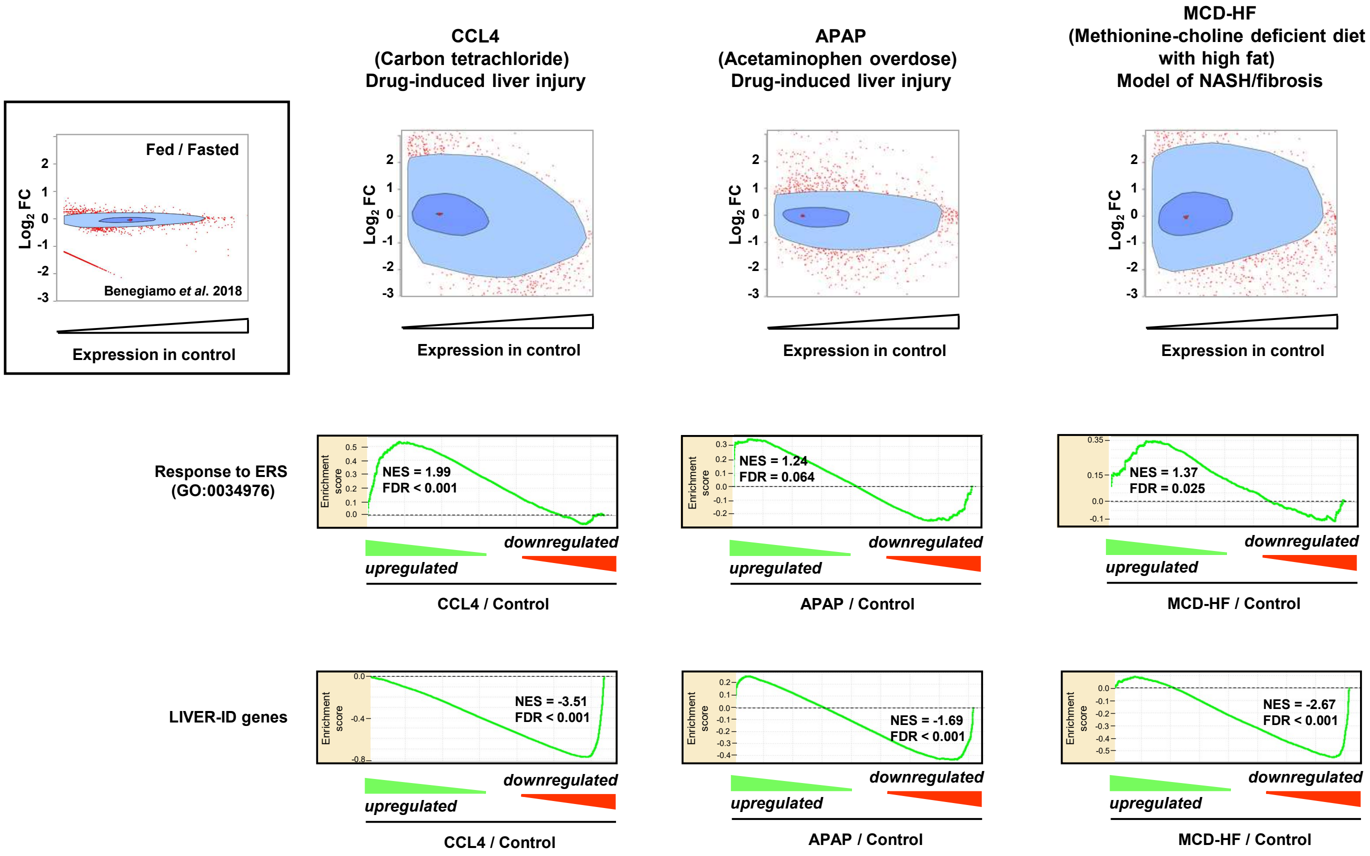

Appendix Figure S20. Analyses of the transcriptomic alterations occurring in additional mouse liver injury models - related to Fig.6. Analyses similar to those performed in Fig.6A-C using transcriptomic data from the indicated additional mouse liver injury models (details are provided in Table EV6). *Inset*, bagplot from Fig.1C provided as a reference.

Appendix Figure S21

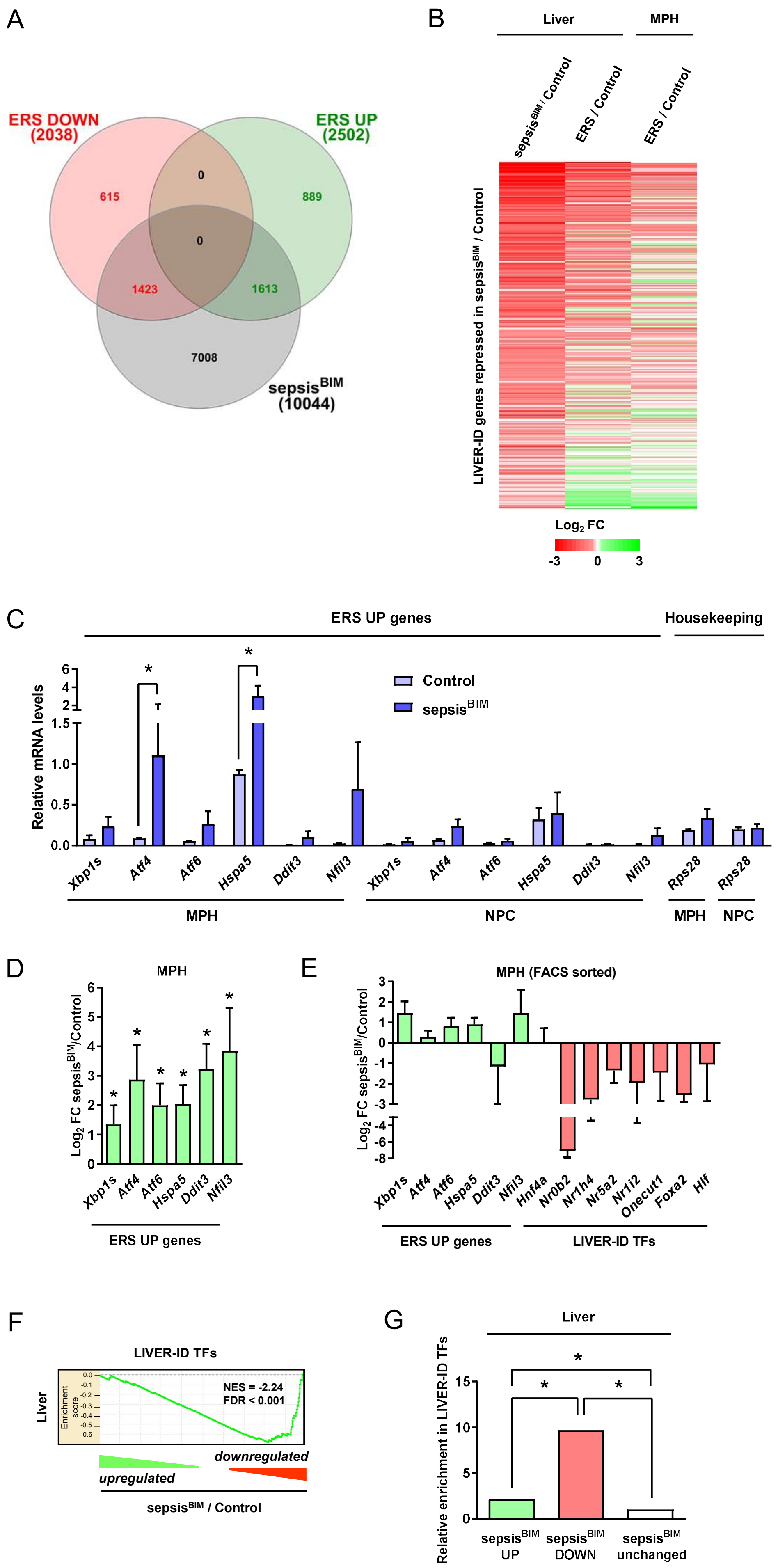

Appendix Figure S21. ERS gene induction and loss of LIVER-ID TFs in the sepsis^{BIM} model - related to Fig.6. (A) Venn diagram showing the overlap between genes significantly modulated by ERS (Fig.1 and Appendix Fig.S2A) and sepsis (Fig.6 and Appendix Fig.S19A). Overall, around 2/3 of ERS-regulated genes are also significantly modulated in septic livers. **(B)** Heatmap showing expression changes (Log_2 FC) of LIVER-ID genes repressed in sepsis^{BIM} mouse liver (332 genes) for the indicated experimental models. Genes were ranked according to the mean Log_2 FC of the 3 datasets. For visualization, Log_2 FC values were saturated at -3 for negative values <-3 and at 3 for positive values >3. **(C)** RT-qPCR analyses of indicated ERS UP genes in MPH and NPC isolated from sepsis^{BIM} and Control mouse livers (3 mice per group). Comparable expression of the *Rps28* housekeeping gene is shown as an additional control. Two-way ANOVA with Bonferroni's post-hoc test was used to assess statistical significance. **(D)** RT-qPCR analyses of selected ERS UP genes in MPH isolated from sepsis^{BIM} and Control mouse livers (5 mice per group). One-sample t-test with BH correction for multiple testing was used to determine if the mean Log_2 FC sepsis^{BIM} vs Control is statistically different from 0. **(E)** RT-qPCR analyses of selected ERS UP genes and LIVER-ID TFs in MPH isolated from sepsis^{BIM} and Control mouse livers and further purified via fluorescence activated cell sorting (FACS) (2 mice per group). **(F)** Enrichment plot from GSEA performed using LIVER-ID TFs as the gene set and transcriptomic changes in sepsis^{BIM} vs Control mouse liver as the ranked gene list. **(G)** The fraction of LIVER-ID TFs among the upregulated and downregulated genes in sepsis^{BIM} vs Control mouse liver is indicated relative to the fraction of LIVER-ID TFs among the genes not statistically significantly affected in sepsis^{BIM} arbitrarily set to 1. Fisher's exact test with BH correction for multiple testing was used to assess statistical significance.

Appendix Figure S22

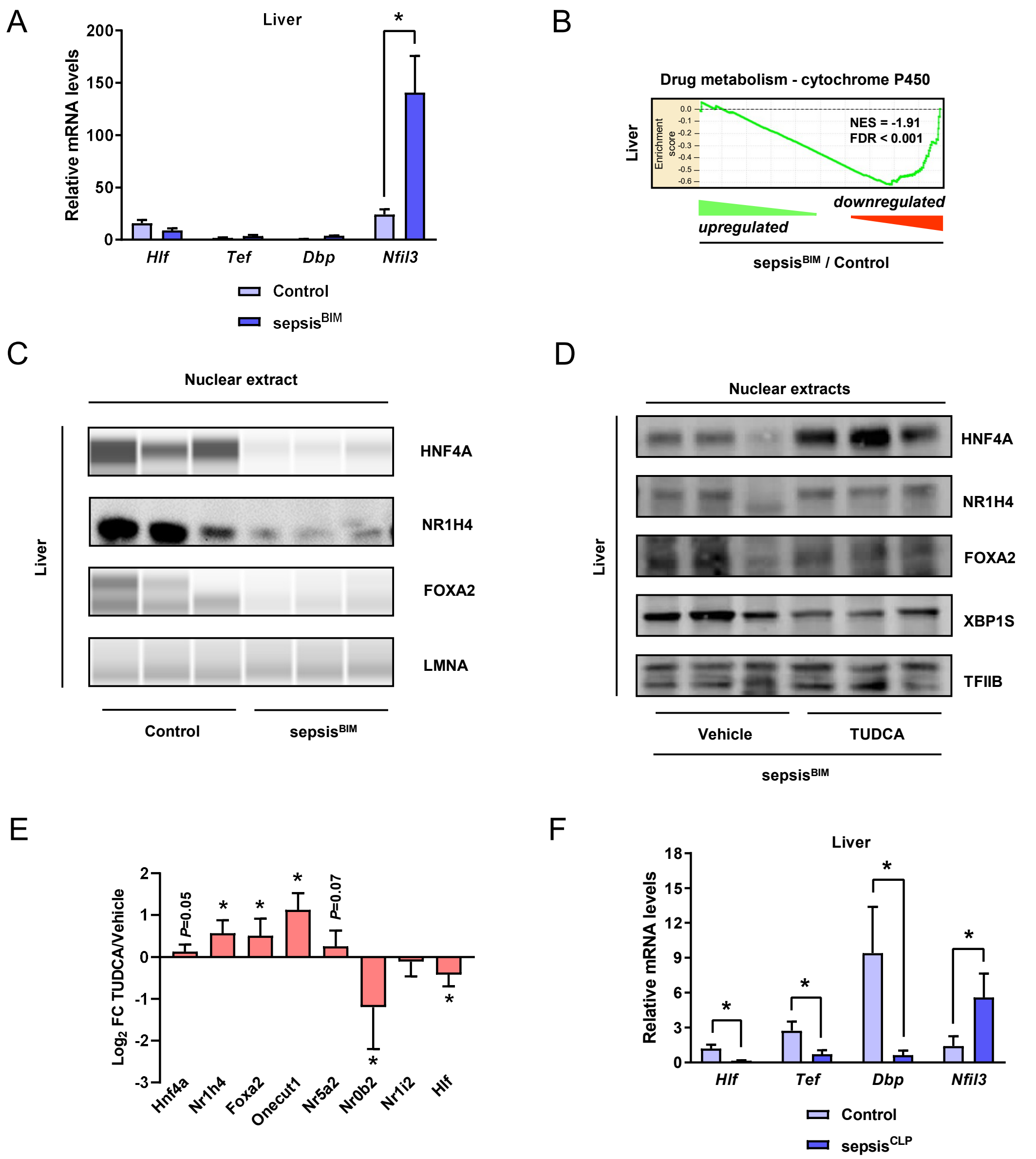

Appendix Figure S22. Expression switch of the PAR-bZIP TF family members in the liver of the sepsis^{BIM} and sepsis^{CLP} mouse models and additional data on LIVER-ID TF expression in the liver of sepsis^{BIM} mice pretreated with TUDCA - related to Fig.6-7. (A) RT-qPCR analyses of *Hlf*, *Tef*, *Dbp* and *Nfil3* expression in Control and sepsis^{BIM} mouse livers (6 mice per group). Two-way ANOVA with Bonferroni's post-hoc test was used to assess statistical significance. **(B)** Enrichment plot from GSEA performed using genes from the "Drug metabolism - cytochrome P450 pathway" (KEGG00982) as the gene set and transcriptomic changes in sepsis^{BIM} vs Control mouse liver as the ranked gene list. **(C)** Western blots similar to those described in Fig.6G using livers from mice sacrificed 6h after bacterial injection, which is the time point used in the TUDCA experiment. **(D)** Western blots similar to those described in Fig.6H using livers from 3 additional mice for each experimental condition. **(E)** RT-qPCR analyses of selected LIVER-ID TFs in livers of sepsis^{BIM} mice pre-treated for 4 consecutive days with vehicle or 500mpk TUDCA (10 mice per group). One-sample t-test with BH correction for multiple testing was used to determine if the mean Log2 FC TUDCA/Vehicle is statistically different from 0. **(F)** RT-qPCR analyses of *Hlf*, *Tef*, *Dbp* and *Nfil3* expression in Control and sepsis^{CLP} mouse livers (15 mice per group). Wilcoxon test with BH correction for multiple testing was used to assess statistical significance.

Supplemental Figure S23

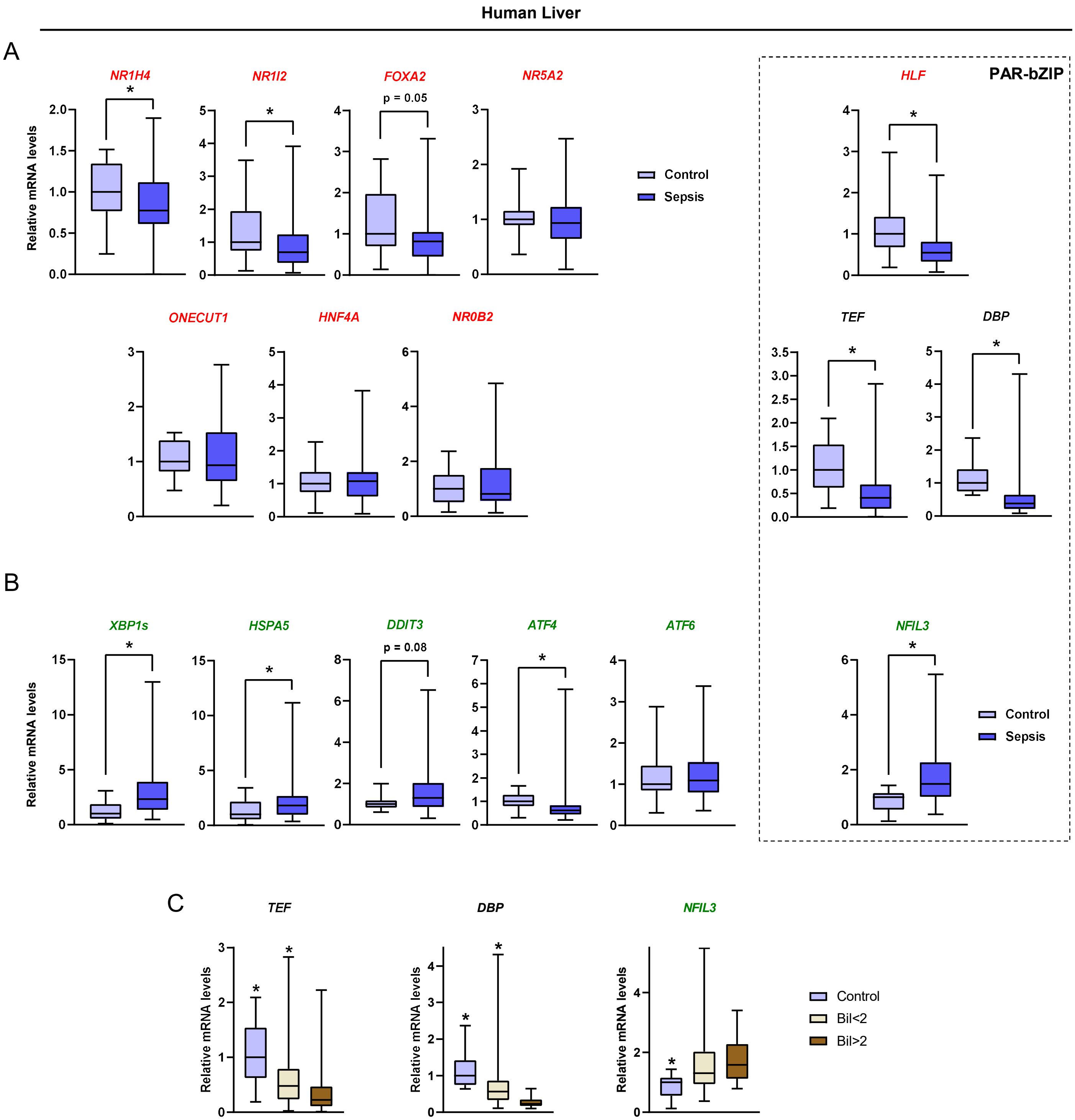

Supplemental Figure S23. Concomitant ERS gene induction and repression of LIVER-ID TF expression in the septic liver of deceased humans - related to Fig.7. (A-B) RT-qPCR analyses of selected LIVER-ID TFs (A) and ERS UP genes (B) monitoring expression changes in the livers of deceased critically-ill patients with sepsis (n=64) *vs* control donors (n=18). PAR-bZIP TF family members are highlighted using the dashed box. ERS UP genes are depicted in green, LIVER-ID TFs in red. Data are shown as box plots, with mRNA levels of the critically-ill group expressed relative to those of the control group. Wilcoxon test was used to assess statistical significance. **(C)** RT-qPCR analyses of the indicated PAR-bZIP TF encoding genes monitoring expression in the livers of Bil<2 (n=34) or Bil>2 (n=28) groups of deceased critically-ill patients with sepsis *vs* control donors (n=18). Data are shown as box plots, with mRNA levels of the critically-ill groups expressed relative to those of the control group. Wilcoxon test was used to assess statistically significant differences with the Bil>2 group.

Appendix Figure S24

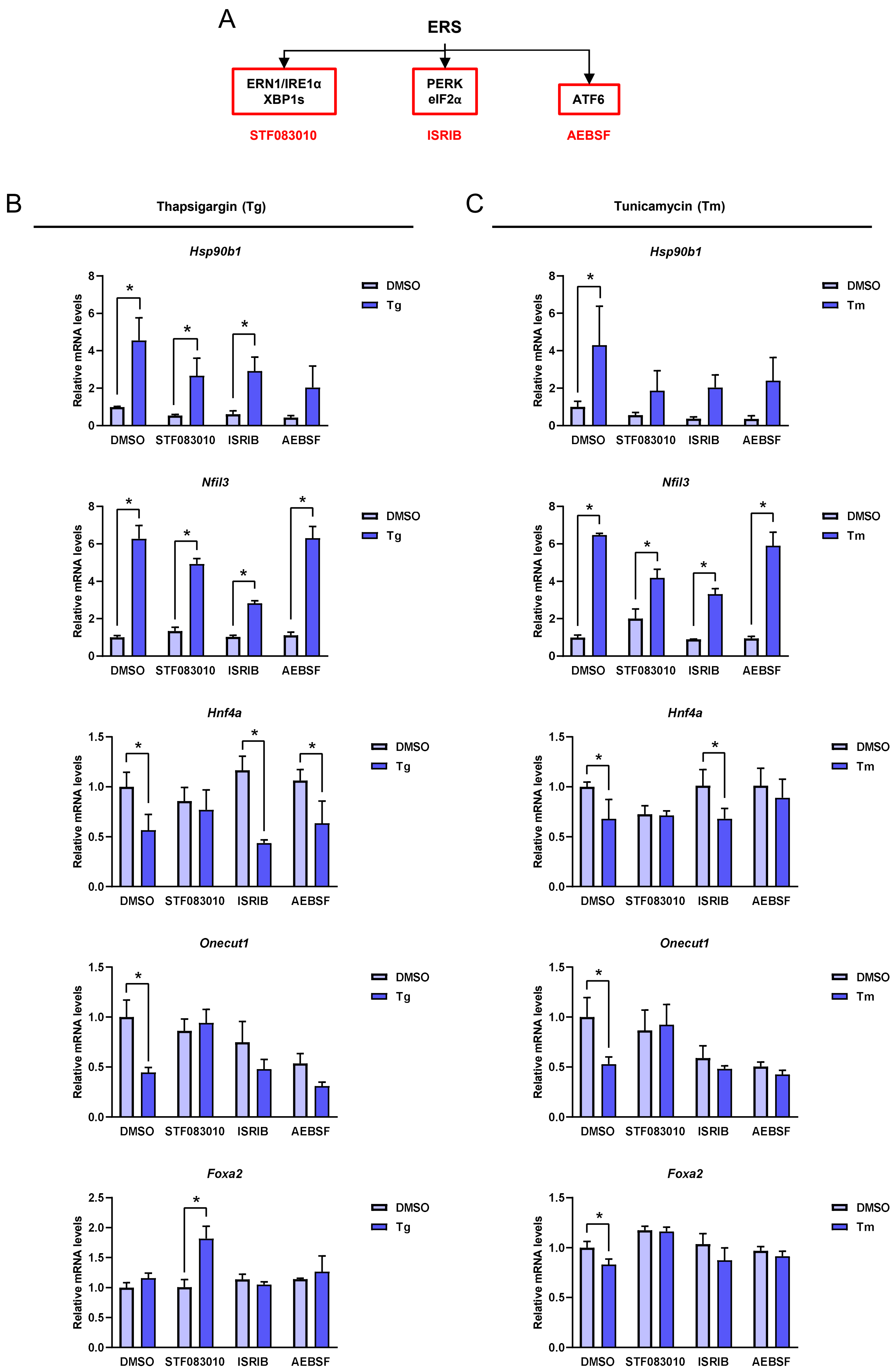

Appendix Figure S24. Effects of inhibitors of ERS sensors induced signaling on LIVER-ID gene repression and *Nfil3* induction - related to Discussion**. (A)** Drugs used to inhibit the three arms of the UPR are indicated in red beneath their respected targeted sensors/signaling pathways. **(B-C)** RT-qPCR analyses of selected ERS UP genes (*Hsp90b1*, *Nfil3*) and LIVER-ID TFs (*Hnf4a*, *Onecut1*, *Foxa2*) in AML12 cells pre-exposed to 30 µM STF083010, 200 µM ISRIB or 100 µM AEBSF for 2h and subsequently treated for 4h with vehicle (Control) or 1µM thapsigargin (*left panel*) or 2µg/mL tunicamycin (*right panel*) (ERS) (3 independent experiments). Two-way ANOVA with Bonferroni's post-hoc test was used to assess statistical significance.

Appendix Figure S25

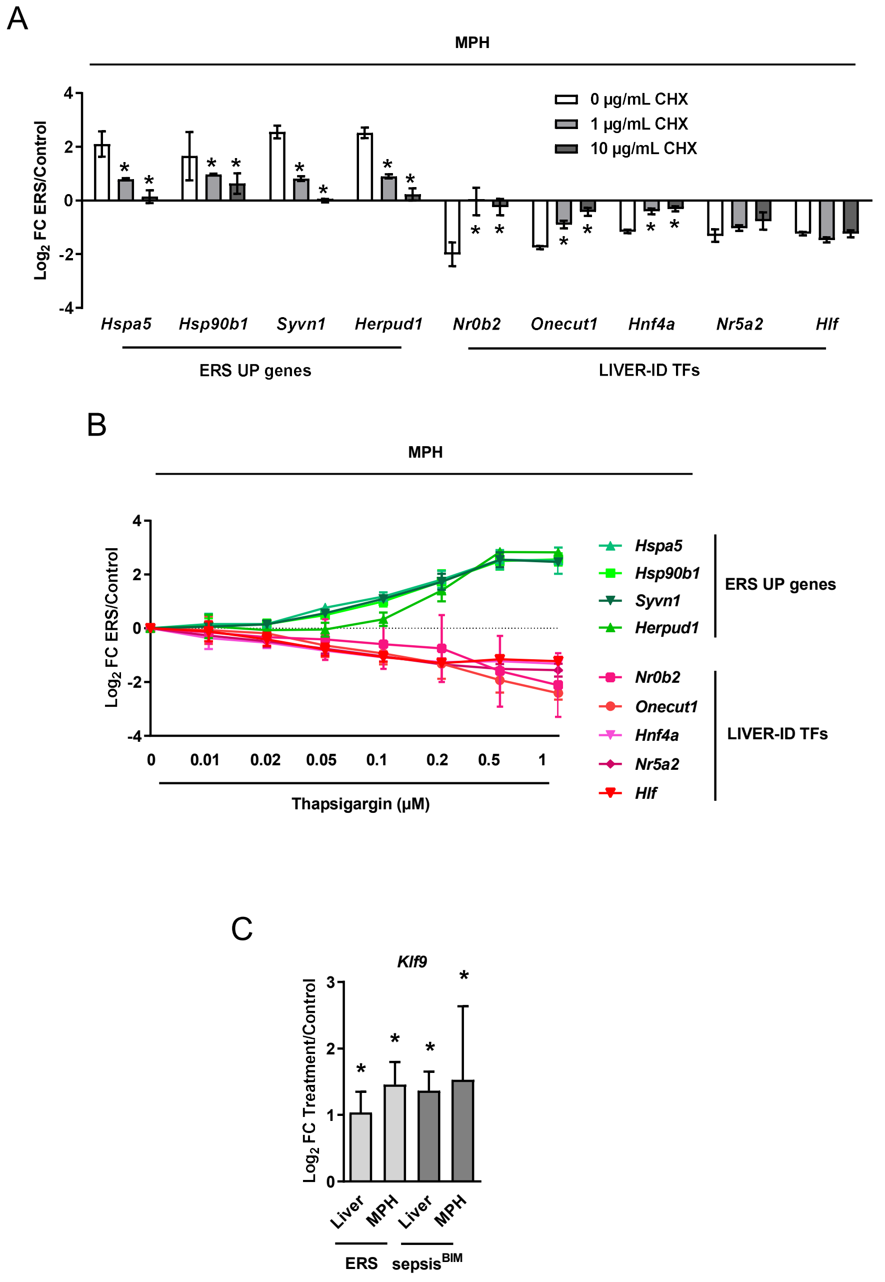

Appendix Figure S25. Additional controls verifying the specificity and relevance of our *in-vitro* acute ERS model in MPH - related to Discussion**. (A)** RT-qPCR analyses of selected ERS UP genes and LIVER-ID TFs in MPH co-treated for 4h with 0, 1 or 10 µg/mL cycloheximide (CHX) and 1µM thapsigargin (ERS) (3 independent experiments). Mean Log_2 FC ERS/Control is shown for every CHX dose. Two-way ANOVA with Dunnett's multiple comparison test was used to assess statistical significance (using 0 µg/mL CHX as the reference). **(B)** RT-qPCR analyses of selected ERS UP genes and LIVER-ID TFs in MPH treated for 4h with vehicle (Control) or increasing thapsigargin doses (ERS) as indicated (3 independent experiments). **(C)** RT-qPCR analyses of *Klf9* expression monitoring changes induced by acute ERS in mouse liver (n=4) or MPH (n=5) (light grey bars) or in sepsis^{BIM} vs Control mouse liver (n=5) or MPH (n=5) (dark grey bars). One-sample t-test with BH correction for multiple testing was used to determine if the mean Log_2 FC Treatment vs Control is statistically different from 0.

Appendix Figure S26

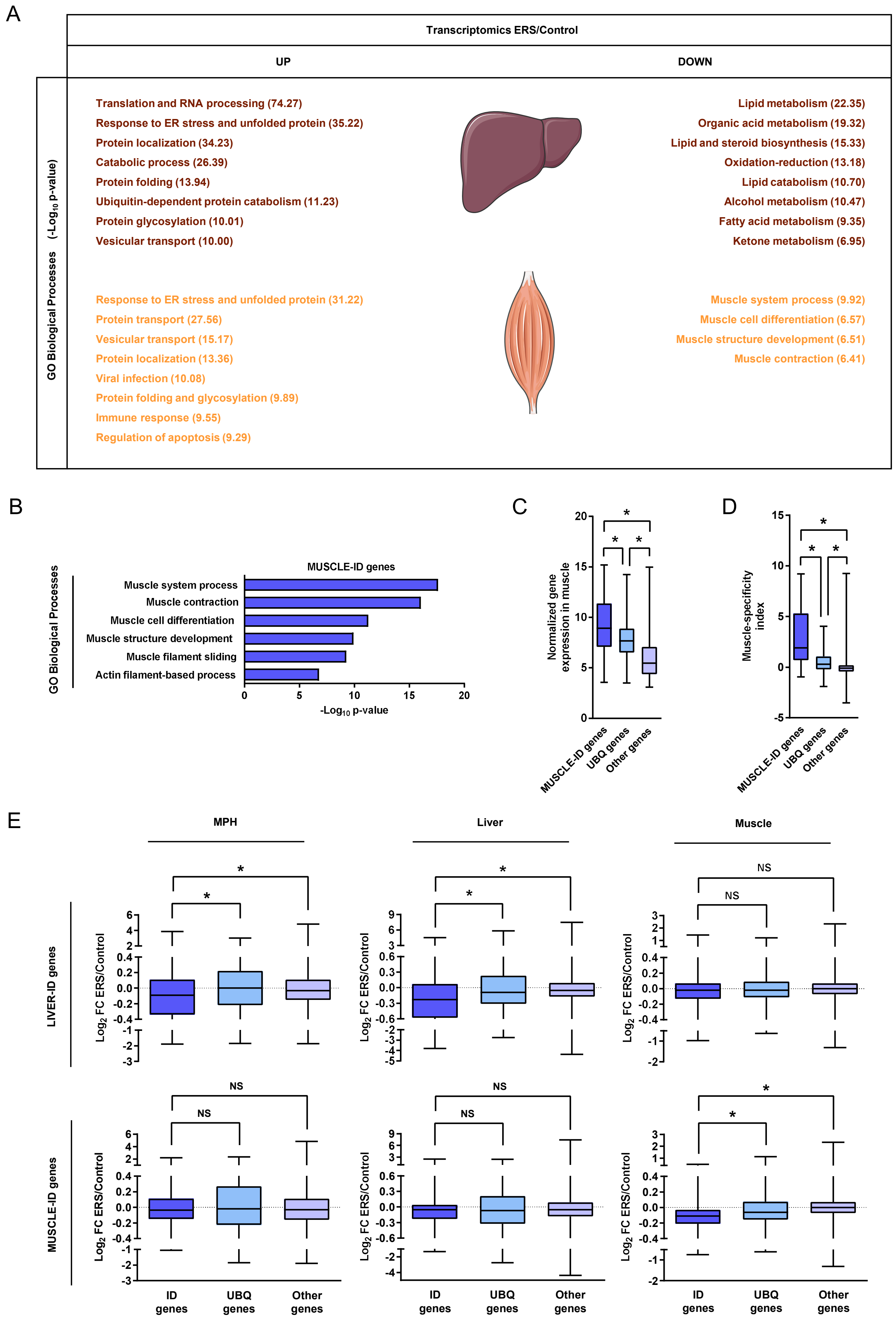

Appendix Figure S26. Loss of molecular identity upon acute ERS is also observed in skeletal muscle - related to Discussion. To assess whether ERS would similarly impede on the identity of another organ, we analyzed the transcriptome of the gastrocnemius skeletal muscles of mice subjected to a single intramuscular injection of tunicamycin or control saline solution in the contralateral leg. **(A)** Functional enrichment analyses were performed using ERS UP and ERS DOWN genes from mouse liver (*upper panels*) or skeletal muscle (*lower panels*) and the ToppGene Suite. GO Biological processes with Bonferroni-corrected p-value $<10^{-6}$ were considered and similar terms were merged. Bonferronicorrected p-values ($-Log_{10}$) are shown. **(B)** Functional enrichment analyses were performed using MUSCLE-ID genes (defined using a strategy similar to the one described for the liver) and the ToppGene Suite. GO Biological processes with Bonferroni-corrected p-value $<10^{-6}$ were considered and similar terms were merged. Bonferroni-corrected p-values ($-Log_{10}$) are shown. MUSCLE-ID and UBQ genes were defined as described in Materials and Methods and are listed in Table EV4. **(C-D)** Box plots showing normalized expression in skeletal muscle (C) and muscle-specificity index (D) for MUSCLE-ID genes, UBQ genes and other genes. Muscle-specificity index was calculated as the difference of normalized expression in skeletal muscle and mean of normalized expression in control tissues using BioGPS data (Table EV6) and is reported as Log_2. One-way ANOVA with Welch's correction and Dunnett's Modified Tukey-Kramer pairwise multiple comparison test was used to assess statistical significance. **(E)** Box plots showing Log_2 FC ERS/Control in MPH (*left panels*), mouse liver (*middle panels*) or mouse skeletal muscle (*right panels*) for ID genes, UBQ genes and other genes. ID genes correspond to LIVER-ID genes (*upper panels*) or MUSCLE-ID genes (*lower panels*). Reminiscent of our findings in liver, the data presented in this figure indicates that ERS led to specific down-regulation of muscle-identity genes. One-way ANOVA with Welch's correction and Dunnett's Modified TukeyKramer pairwise multiple comparison test was used to assess statistical significance. NS = not significant.

Appendix Figure S27

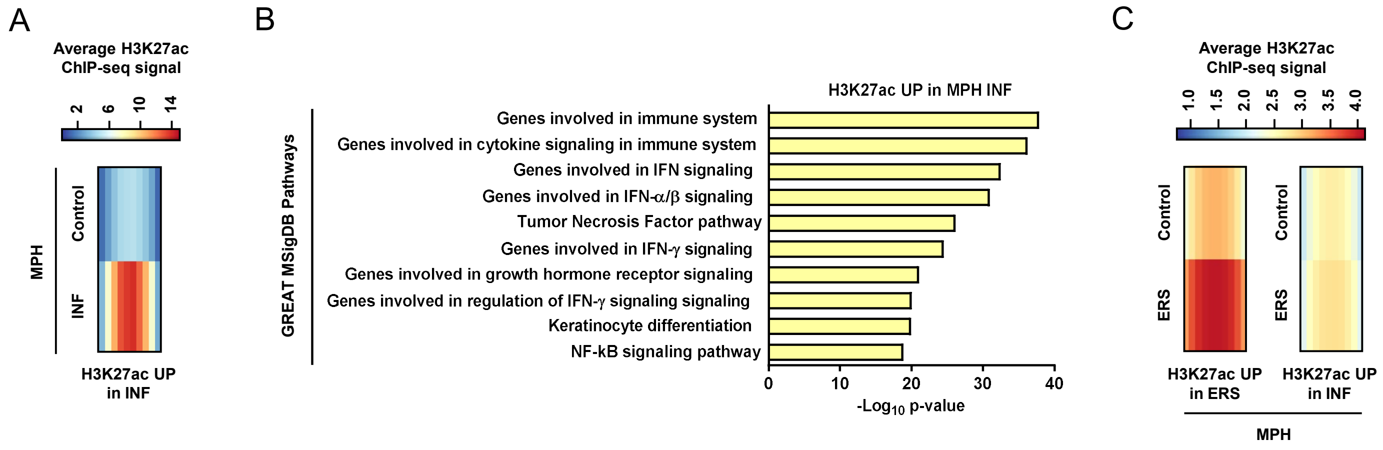

Appendix Figure S27. Acute ERS in MPH does not trigger activation of CRMs associated with an inflammatory response - related to Discussion. (A) Heatmap showing the average H3K27ac ChIP-seq signal for regions with increased H3K27ac in MPH subjected to an inflammatory stimulus (INF, combination of 10 ng/mL IL-1β and 10 ng/mL IL-6 for 2h). Data are means from 3 independent experiments reported in (Goldstein et al., 2017), which were lifted to mm10 using the Galaxy Liftover tool. **(B)** Functional enrichment analyses performed using the H3K27ac regions with increased levels upon INF from A and Genomic Regions Enrichment of Annotations Tool (GREAT 3.0.0) (default parameters) (McLean et al., 2010). FDR corrected p-values (-Log_{10}) are shown for the 10 top ranked MSigDB Pathways. **(C)** Heatmaps showing the average H3K27ac ChIP-seq signals in MPH treated with vehicle (Control) or 1µM thapsigargin (ERS) for regions with increased H3K27ac in MPH subjected to ERS (*left*, regions from Fig.2) and for regions with increased H3K27ac in MPH subjected to inflammation (INF) (*right*, regions from panel A). These data show that acute ERS in MPH does not lead to increased H3K27ac levels within regions involved in hepatic inflammatory response indicating repression of LIVER-ID genes by ERS cannot be ascribed to indirect induction of an inflammatory phenotype.

APPENDIX REFERENCES

D'Alessio Ana C, Fan Zi P, Wert Katherine J, Baranov P, Cohen Malkiel A, Saini Janmeet S, Cohick E, Charniga C, Dadon D, Hannett Nancy M, Young Michael J, Temple S, Jaenisch R, Lee Tong I, Young Richard A (2015) A Systematic Approach to Identify Candidate Transcription Factors that Control Cell Identity. *Stem Cell Reports* 5: 763-775

Goldstein I, Paakinaho V, Baek S, Sung M-H, Hager GL (2017) Synergistic gene expression during the acute phase response is characterized by transcription factor assisted loading. *Nature Communications* 8: 1849

McLean CY, Bristor D, Hiller M, Clarke SL, Schaar BT, Lowe CB, Wenger AM, Bejerano G (2010) GREAT improves functional interpretation of cis-regulatory regions. *Nat Biotechnol* 28: 495-501

Zhou Q, Liu M, Xia X, Gong T, Feng J, Liu W, Liu Y, Zhen B, Wang Y, Ding C, Qin J (2017) A mouse tissue transcription factor atlas. *Nat Commun* 8: 15089
